# Aging and high-fat diet feeding lead to peripheral insulin resistance and sex-dependent changes in brain of mouse model of tau pathology THY-Tau22

**DOI:** 10.1186/s12974-021-02190-3

**Published:** 2021-06-22

**Authors:** Miroslava Kacířová, Blanka Železná, Michaela Blažková, Martina Holubová, Andrea Popelová, Jaroslav Kuneš, Blanka Šedivá, Lenka Maletínská

**Affiliations:** 1 Institute of Organic Chemistry and Biochemistry of the Czech Academy of Sciences, Flemingovo náměstí 542/2, 160 00 Prague 6, Czech Republic; 2 Institute of Physiology of the Czech Academy of Sciences, Vídeňská 1083, 142 20 Prague 4, Czech Republic; 3 Department of Mathematics, University of West Bohemia, Univerzitní 2732/8, 301 00 Pilsen, Czech Republic

**Keywords:** Alzheimer’s disease, THY-Tau22 mouse, Obesity, Neuroinflammation, Peripheral insulin resistance, Sex differences

## Abstract

**Background:**

Obesity leads to low-grade inflammation in the adipose tissue and liver and neuroinflammation in the brain. Obesity-induced insulin resistance (IR) and neuroinflammation seem to intensify neurodegeneration including Alzheimer’s disease. In this study, the impact of high-fat (HF) diet-induced obesity on potential neuroinflammation and peripheral IR was tested separately in males and females of THY-Tau22 mice, a model of tau pathology expressing mutated human tau protein.

**Methods:**

Three-, 7-, and 11-month-old THY-Tau22 and wild-type males and females were tested for mobility, anxiety-like behavior, and short-term spatial memory in open-field and Y-maze tests. Plasma insulin, free fatty acid, cholesterol, and leptin were evaluated with commercial assays. Liver was stained with hematoxylin and eosin for histology. Brain sections were 3′,3′-diaminobenzidine (DAB) and/or fluorescently detected for ionized calcium-binding adapter molecule 1 (Iba1), glial fibrillary acidic protein (GFAP), and tau phosphorylated at T231 (pTau (T231)), and analyzed. Insulin signaling cascade, pTau, extracellular signal-regulated kinase 1/2 (ERK1/2), and protein phosphatase 2A (PP2A) were quantified by western blotting of hippocampi of 11-month-old mice. Data are mean ± SEM and were subjected to Mann-Whitney t test within age and sex and mixed-effects analysis and Bonferroni’s post hoc test for age comparison.

**Results:**

Increased age most potently decreased mobility and increased anxiety in all mice. THY-Tau22 males showed impaired short-term spatial memory. HF diet increased body, fat, and liver weights and peripheral IR. HF diet-fed THY-Tau22 males showed massive Iba1+ microgliosis and GFAP+ astrocytosis in the hippocampus and amygdala. Activated astrocytes colocalized with pTau (T231) in THY-Tau22, although no significant difference in hippocampal tau phosphorylation was observed between 11-month-old HF and standard diet-fed THY-Tau22 mice. Eleven-month-old THY-Tau22 females, but not males, on both diets showed decreased synaptic and postsynaptic plasticity.

**Conclusions:**

Significant sex differences in neurodegenerative signs were found in THY-Tau22. Impaired short-term spatial memory was observed in 11-month-old THY-tau22 males but not females, which corresponded to increased neuroinflammation colocalized with pTau(T231) in the hippocampi and amygdalae of THY-Tau22 males. A robust decrease in synaptic and postsynaptic plasticity was observed in 11-month-old females but not males. HF diet caused peripheral but not central IR in mice of both sexes.

**Supplementary Information:**

The online version contains supplementary material available at 10.1186/s12974-021-02190-3.

## Introduction

The prevalence of obesity is increasing worldwide [1], leading to diverse comorbidities and increasing the cost of their cure. Obesity can cause insulin resistance (IR) not only in the periphery but also in the brain. IR is characterized by reduced sensitivity of insulinotropic tissues to the action of insulin and is one of the major pathophysiological attributes of type 2 diabetes mellitus [2]. In addition, obesity supports low-grade inflammation in the adipose tissue and the liver and neuroinflammation in the hypothalamus [2, 3]. Recently, obesity-related neuroinflammation has been detected in extrahypothalamic brain structures such as the hippocampus or brain cortex (reviewed by [3, 4]) and linked to impaired cognitive function. Both IR and low-grade neuroinflammation resulting from obesity seem to intensify neurodegenerative changes in brains affected by Alzheimer’s disease (AD) [5].

In addition to beta-amyloid (Aβ) plaques, hyper and abnormally phosphorylated tau protein [6], which is prone to form neurofibrillary tangles and finally to cause synapse failure, is another main AD hallmark. The transgenic model THY-Tau22 was established to elucidate the role of tau pathology and related pathological changes in the brains of patients with AD and other tauopathies [7]. THY-Tau22 mice overexpress human four-repeat tau with G272V and P301S mutations under the Thy1.2 promoter, mainly in the brain, and therefore are nearly free of motor dysfunctions. These mice develop tau hyper and abnormal phosphorylation, neurofibrillary tangles, loss of functional synapses, astrogliosis, and mild cognitive impairment, progressing with age [7–9].

In THY-Tau22 mice, a potential interrelation between tau pathology and astroglial and microglial immune responses was shown and linked to an early chemokine response that attracted T cells into the hippocampus [10]. Furthermore, the increasing presence of hippocampal CD11b (integrin αM) with increasing age was observed in the THY-Tau22 mouse model. In addition, the mRNA expression of innate immunity markers *Tlr2* (Toll-like receptor 2), *CD68* (cluster of differentiation 68), and *TNFα* (tumor necrosis factor α) was found to be increased [10]. This finding points to a fundamental role of neuroinflammation in tau pathology.

Neuroinflammation, represented by an increase in reactive glial cells and cytokine activation, was also found in the hypothalami of insulin-deficient rodents [11] and those with high-fat (HF) diet-induced obesity and in obese humans [12]. HF diet-induced obesity in rodents was reported to cause IR that mostly cohered with enhanced tau phosphorylation [13, 14], mainly in the hippocampus. However, others found neither tau hyperphosphorylation nor neuroinflammation in the hippocampi and brain cortex of male Wistar rats fed a high-fat/high-sucrose diet [15]. Moreover, HF diet-induced obesity—but neither peripheral nor central IR—was specified as the reason for hippocampal tau hyperphosphorylation and worsened learning ability in THY-Tau22 mice; however, no link of HF diet-induced obesity to neuroinflammation was mentioned in the study [16]. Furthermore, Mangold et al. found sexually divergent neuroinflammation with aging in C57BL6 mice that might contribute to sex differences in age-related neurological diseases such as stroke and Alzheimer’s disease, specifically in the complement system. They found that age-related hippocampal induction of neuroinflammatory gene expression was sexually divergent and enriched for microglia-specific genes such as complement pathway components. It seems that age is the major cause of difference in neuroinflammation between sexes [17].

The present study aims to determine if age-dependent obesity resulting from HF diet feeding causes IR in the periphery, affects insulin signaling in the hippocampus, leads to impaired age-related behavioral and memory changes, and worsens neuroinflammation in the brain separately in males and females of THY-Tau22 mice and corresponding wild-type (wt) controls. Additionally, we examine whether these two pathologies—potential neuroinflammation resulting from obesity and pathological tau—colocalize in brains of THY-Tau22 mice and corresponding wt controls.

## Materials and methods

### Animals

THY-Tau22 male and female mice with a C57Bl/6 J background, generous gift of Dr. Luc Buée (Lille Neuroscience & Cognition, Inserm UMR1172, France), were genotyped [7] and bred at the Biotechnology and Biomedicine Centre of the Academy of Sciences and Charles University (BIOCEV) (Vestec, Czech Republic). From the age of 7 weeks, the mice were housed and handled in the animal facility of the Institute of Organic Chemistry and Biochemistry of the Czech Academy of Sciences, Prague, Czech Republic, with a 12-h light/dark cycle (lights on at 6 a.m.) and temperature set at 23 ± 2 °C. Wt C57Bl/6 J mice of both sexes were used as controls. Mice were housed 4–6 per cage with ad libitum access to water and food (Table 1). The wt and THY-Tau22 groups of both sexes sacrificed at 3 months (n = 4–5) were fed a standard (St) diet (standard rodent diet Ssniff® R/M-H (Ssniff Spezialdiäten GmbH, Soest, Germany)). The wt and THY-Tau22 mice of both sexes sacrificed at 7 (n = 7–8) or 11 (n = 7–14) months of age were fed either a St or HF diet. Feeding the HF diet started from 8 weeks of age. The caloric content percentage values for the HF diet were 13% proteins, 60% fats, and 27% carbohydrates [18]. All mice were weighed once per week when the HF diet feeding started until the day of dissection. The age of mice sacrification was designed based on the previous model characterization where the particular signs of neurodegeneration were established [7, 9].

**Table 1:** Experimental study design time course of THY-Tau22 and wt

Age			3 months		7 months			11 months		
Weeks on diet	0	1	6	8	22	23	24	37	38	42
**Diet, treatment**	St	St	Behavioral tests	Dissection after 12 h fasting	–	–	–	–	–	–
**Diet, treatment**	St	St/HF	–	–	OGTT after 6 h fasting	Behavioral tests	Dissection after 12 h fasting	–	–	–
**Diet, treatment**	St	St/HF	–	–	–	–	–	OGTT after 6 h fasting	Behavioral tests	Dissection after 12 h fasting

Week 0 on diet corresponds to 7 weeks of age

*Abbreviation*s: *HF* high-fat diet, *OGTT* oral glucose tolerance test, *St* standard chow diet

The animal experiments followed the ethical guidelines for animal experiments in the Czech Republic Act Nr. 246/1992 and were approved by the Committee for Experiments with Laboratory Animals of the Czech Academy of Sciences.

### Behavioral tests

Behavioral tests were performed 2 weeks before the dissection (Table 1).

#### Open field

Anxiety-like behavior, exploration, and locomotion were tested by the open field behavioral test [19], which was performed in a 50× 50× 50 cm^3^ box. The mouse was placed into the middle part of the box, and the recording started immediately after placement and lasted for 10 min. The obtained record was analyzed using EthoVision XT software (Noldus, Wageningen, Netherlands). The average velocity and wall distance were monitored. The average velocity includes the mouse behavior including grooming and freezing.

#### Y-maze

Short-term spatial memory was tested by a Y-maze behavioral test described previously [20]. The obtained records were analyzed using EthoVision XT software.

The average time spent in the new zone (NZ) and the average number of entries into the NZ at the second session of the experiment were monitored.

### Oral glucose tolerance test

One week before behavioral tests (Table 1), 7- and 11-month-old mice were tested for tolerance to glucose with an oral glucose tolerance test (OGTT). After 6 h of fasting, the concentration of blood glucose was measured in the blood from the tip of the tail using a Glucocard^TM^ X-meter (Arkray factory, Inc., Japan) as described previously [21]. Briefly, glucose at a dose of 2 g/kg of mouse body weight was administered by gavage. Blood glucose concentration was determined at 15, 30, 60, 90, 120, and 180 min after glucose gavage.

The time curves of ΔGlucose were plotted; ΔGlucose = c_Glc_(t) – c_Glc_(t_0_), where c_Glc_(t) is the concentration of glucose at a particular time t and c_Glc_(t_0_) is the concentration of glucose at time 0 min. The areas under the curves (AUC_Δglucose_) were calculated using GraphPad Prism 8.

### Blood plasma inflammation marker

The concentration of C-reactive protein (CRP) in non-anesthetized fasting plasma was determined using Mouse CRP ELISA kit (Thermo Scientific, Frederick, MD, USA). The CRP was determined in 11-month-old mice of both sexes. The measurement was performed according to manufacturer’s instructions.

### Organ dissection

Blood samples from the overnight fasád animals were collected from the tail veins, and the concentrations of blood glucose were measured. The blood plasma was separated with EDTA and stored at − 80 °C. Then, the mice were deeply anesthetized with pentobarbital (170 mg/kg of mouse body weight, Sigma Aldrich, St. Louis, MO, USA), the left heart ventricle was punctured, blood was collected, and EDTA plasma was isolated and stored at − 80 °C. The mice were transcardially perfused with saline solution supplemented with heparin (10 U/ml, Zentiva, Prague, Czech Republic).

Brains were dissected on ice. The right hemispheres of each brain were postfixed in 4% paraformaldehyde (PFA) as described previously [22] and stored in a 30% sucrose solution with the addition of sodium azide until cutting for immunohistochemistry (IHC). The hippocampi from the left hemispheres were covered with 200 μl of ice-cold lysis buffer (62.5 mM TRIS-HCl pH 6.8, 1% Triton X-100, 1% deoxycholate, 50 mM NaF, 1 mM Na_3_VO_3_, cOmplete^TM^ Protease Inhibitor Cocktail (CO-RO) (1 tablet/50 ml of solution), Roche, Mannheim, Germany) and stored at − 80 °C until homogenization for western blots (WB).

The subcutaneous adipose tissue (SCAT) and intraperitoneal adipose tissue (IPAT) were removed and weighed.

The liver was weighed and immersed in 4% PFA, and after 24 h, it was stored at 4 °C in 70% ethanol, embedded into paraffin blocks, and stored until cutting for histology.

### Biochemical parameters

The concentration of fasting plasma insulin was measured by RIA assay (Millipore, St. Charles, MI, USA). The concentration of free fatty acids (FFA) in plasma samples was determined using a colorimetric assay (Roche, Mannheim, Germany). The concentration of cholesterol in the plasma samples was determined using a colorimetric assay kit (Erba Lachema, Brno, Czech Republic). Plasma triglyceride (TG) levels were measured using a quantitative enzymatic reaction kit (Sigma, St. Louis, MO, USA). The leptin concentration in plasma samples was determined by ELISA (Millipore, St. Charles, MI, USA). All measurements were performed according to the manufacturer's instructions.

### Insulin resistance quantification

The homeostatic model assessment for insulin resistance (HOMA-IR) and quantitative insulin sensitivity check index (QUICKI) were calculated from the measured fasting plasma glucose and fasting plasma insulin concentrations as described previously [23, 24]. Briefly, HOMA-IR and QUICKI were calculated using the following equations: HOMA-IR = {(fasting plasma glucose) [mmol/l] × (fasting plasma insulin) [pmol/l]}/22.5; QUICKI = 1/[(log(I_0_) + log(G_0_)], where I_0_ is the fasting plasma insulin level [μU/ml], and G_0_ is the fasting blood glucose level [mg/dl].

### Liver histology

The liver slices were stained with hematoxylin and eosin for histological analysis as described previously [4].

### Brain immunohistochemistry

PFA-fixed right brain hemispheres were cut according to Holubová et al. [22]. The sections were kept in cryoprotective solution (30% (v/v) ethylene glycol, 25% (v/v) glycerol, 45% (v/v) pH 7.4 1× PBS) at − 20 °C until immunohistochemistry staining.

3,3′-Diaminobenzidine (DAB) staining with peroxidase rabbit IgG was performed as described previously [22]. Fluorescence staining was performed the same with the following modifications: the primary antibody (Table 2) was diluted in 3% normal goat serum (NGS). In the final step of staining, the sections were washed 2 times with TBS-T and 1 time with 1× TBS, mounted on silanized slides, and coverslipped using Vectashield® Antifade Mounting Medium with 4′,6′-diamidin-2-phenylindole (DAPI) (Vector Laboratories, Inc., Burlingame, CA, USA). The samples were stored in the dark at 4 °C until imaging. The list of secondary antibodies is in Table 3. Fluorescence staining was used for the colocalization of activated astrocytes (glial fibrillary acidic protein, GFAP), phosphorylated tau at the T231 residue (pTau (T231)) (AT180), and cell nuclei (DAPI).

**Table 2:** Primary antibodies used at immunohistochemistry

Primary antibody	Dilution	DAB staining	Manufacturer
GFAP rabbit pAb	1:200	1 min, 10 sec	Thermofisher, Rockford, IL, USA
Iba1 rabbit pAb	1:2000	3 mins	FUJIFILM Wako Pure Chemical Corp., Richmond, VA, USA
AT180 mouse mAb	1:400	-	Thermofisher, Rockford, IL, USA

*DAB* 3,3′-diaminobenzidine, *GFAP* glial fibrillary acidic protein, *Iba1* ionized calcium-binding adapter molecule 1

**Table 3:** Secondary antibodies used at immunohistochemistry

Staining	Secondary antibody	Dilution	Manufacturer
DAB	Peroxidase rabbit IgG	1:400	Vector Lab., Burlingame, CA, USA
Fluorescence	AlexaFluor 488 mouse pAb	1:1000	Thermofisher, Rockford, IL, USA
	AlexaFluor 568 rabbit pAb	1:1000	Thermofisher, Rockford, IL, USA

*DAB* 3,3′-diaminobenzidine

Stained sections were imaged by an IX83 P1ZF Olympus Microscope (Olympus Corporation, Tokyo, Japan) equipped with a DP74 camera with bright field or fluorescence CoolLed pE-Universal Collimator sources using OLYMPUS CellSens Dimension software. Six to seven approximately same size middle-part sections per brain (n = 4 randomly chosen from each group) were analyzed for DAB staining. The area of interest was selected manually according to the mouse brain atlas (The mouse brain in stereotaxic coordinates, Paxinos and Franklin, 1997). Using ImageJ software (NIH, Bethesda, MD, USA), the areas of interest of stained sections were analyzed as a percentage of a dark background from the area of interest (the hippocampus, cortex, and amygdala for ionized calcium-binding adapter molecule 1 (Iba1), and the hippocampus and cortex for GFAP) divided by the average percentage of a dark background from the area of interest of wt mice on the St diet. The threshold was kept constant for all samples in the particular staining experiment. All areas of interests were approximately the same size across the chosen brain sections.

The fluorescence-stained sections were not quantified. Illustrative pictures were chosen for the presentation.

### Western blotting

The hippocampi (n = 6 mice per group) were homogenized in 200 μl of ice-cold lysis buffer using a Bullet Blender homogenizer (Next Advance, Inc. Averill Park, NY, USA). The homogenates were sonicated for 1 min and centrifuged for 8 min at 3080×*g* at 4 °C. The supernatant protein concentrations were determined using a Pierce BCA protein assay kit (Thermo Fisher Scientific, Waltham, MA, USA). The supernatant samples (7 μg of protein/7 μl of sample/line) were prepared and resolved according to Holubová et al. [22]. The list of primary antibodies used is shown in Table 4. Transferred protein bands on nitrocellulose membrane (Bio-Rad, Hercules, CA, USA) were visualized by ChemiDoc^TM^ MP System (Bio-Rad, Hercules, CA, USA) and quantified using Image Lab Software (Bio-Rad, Hercules, CA, USA) as described previously [22].

**Table 4:** Primary antibodies used at western blots of hippocampi

Primary antibody	Dilution	Diluent	Manufacturer
β-actin mouse mAb	1:10,000	5% milk	Sigma-Aldrich, St. Louis, MO, USA
Akt rabbit mAb	1:1000	5% BSA	Cell Signaling Technology, Danvers, MA, USA
pAkt (T308) rabbit mAb	1:1000	5% BSA	Cell Signaling Technology, Danvers, MA, USA
pAkt (S473) rabbit mAb	1:1000	5% BSA	Cell Signaling Technology, Danvers, MA, USA
CD11b rabbit mAb	1:1000	5% milk	Abcam, Cambridge, UK
p44/42 ERK1/2 mouse mAb	1:2000	5% milk	Cell Signaling Technology, Danvers, MA, USA
p44/42 pERK1/2 (T202/Y204) mouse mAb	1:2000	5% milk	Cell Signaling Technology, Danvers, MA, USA
GFAP mouse mAb	1:1000	5% milk	Cell Signaling Technology, Danvers, MA, USA
GSK-3β rabbit mAb	1:1000	5% BSA	Cell Signaling Technology, Danvers, MA, USA
pGSK-3β (S9) rabbit mAb	1:1000	5% BSA	Cell Signaling Technology, Danvers, MA, USA
PDK-1 rabbit pAb	1:1000	5% BSA	Cell Signaling Technology, Danvers, MA, USA
pPDK-1 (S241) rabbit mAb	1:1000	5% BSA	Cell Signaling Technology, Danvers, MA, USA
PI3Kp85 rabbit mAb	1:1000	5% BSA	Cell Signaling Technology, Danvers, MA, USA
PP2A C rabbit mAb	1:1000	5% BSA	Cell Signaling Technology, Danvers, MA, USA
methyl-PP2A C (L309) mouse mAb	1:1000	5% milk/ 1% BSA	Millipore, Temecula, CA, USA
pPP2A (Y307) rabbit mAb	1:5000	5% milk	Abcam, Cambridge, UK
PSD95 rabbit pAb	1:1000	5% BSA	Cell Signaling Technology, Danvers, MA, USA
Synaptophysin rabbit pAb	1:5000	5% milk	Santa Cruz Biotechnology, Inc., Dallas, TX, USA
Tau 5 mouse mAb	1:5000	5% milk	Invitrogen Corp., Frederick, MD, USA
pTau (pThr212) rabbit pAb	1:5000	5% BSA	Thermofisher, Rockford, IL, USA
pTau (pSer214) rabbit pAb	1:1000	5% BSA	Thermofisher, Rockford, IL, USA
pTau (pThr231) rabbit pAb	1:10,000	5% BSA	Thermofisher, Rockford, IL, USA
pTau (pSer396) rabbit pAb	1:10,000	5% BSA	Thermofisher, Rockford, IL, USA
pTau (pSer404) rabbit pAb	1:1000	5% BSA	Thermofisher, Rockford, IL, USA
pTau (pSer422) rabbit pAb	1:1000	5% BSA	Thermofisher, Rockford, IL, USA

*BSA* bovine serum albumin, *mAb* monoclonal antibody, *pAb* polyclonal antibody

All diluents are diluted in TBS/Tween-20; non-fat dry milk was used

### Statistical analyses

The data are presented as the mean ± SEM and were analyzed with GraphPad Prism 8.0 Software (San Diego, CA, USA) using the nonparametric unpaired Mann-Whitney t test for particular pair comparison (wt vs THY-Tau22, St vs HF diet) within each age and sex group and nonparametric unpaired mixed-effects analysis of two-way ANOVA and Bonferroni’s post hoc test for age comparison as stated in the figure and table legends. The area under the curve (AUC_ΔGlucose_) was statistically analyzed using nonparametric unpaired Kruskal-Wallis one-way ANOVA and Bonferroni’s post hoc test within each age and sex group. p < 0.05 was considered statistically significant.

## Results

### The combination of age and a HF diet significantly worsened the mobility of mice

**Fig. 1:**
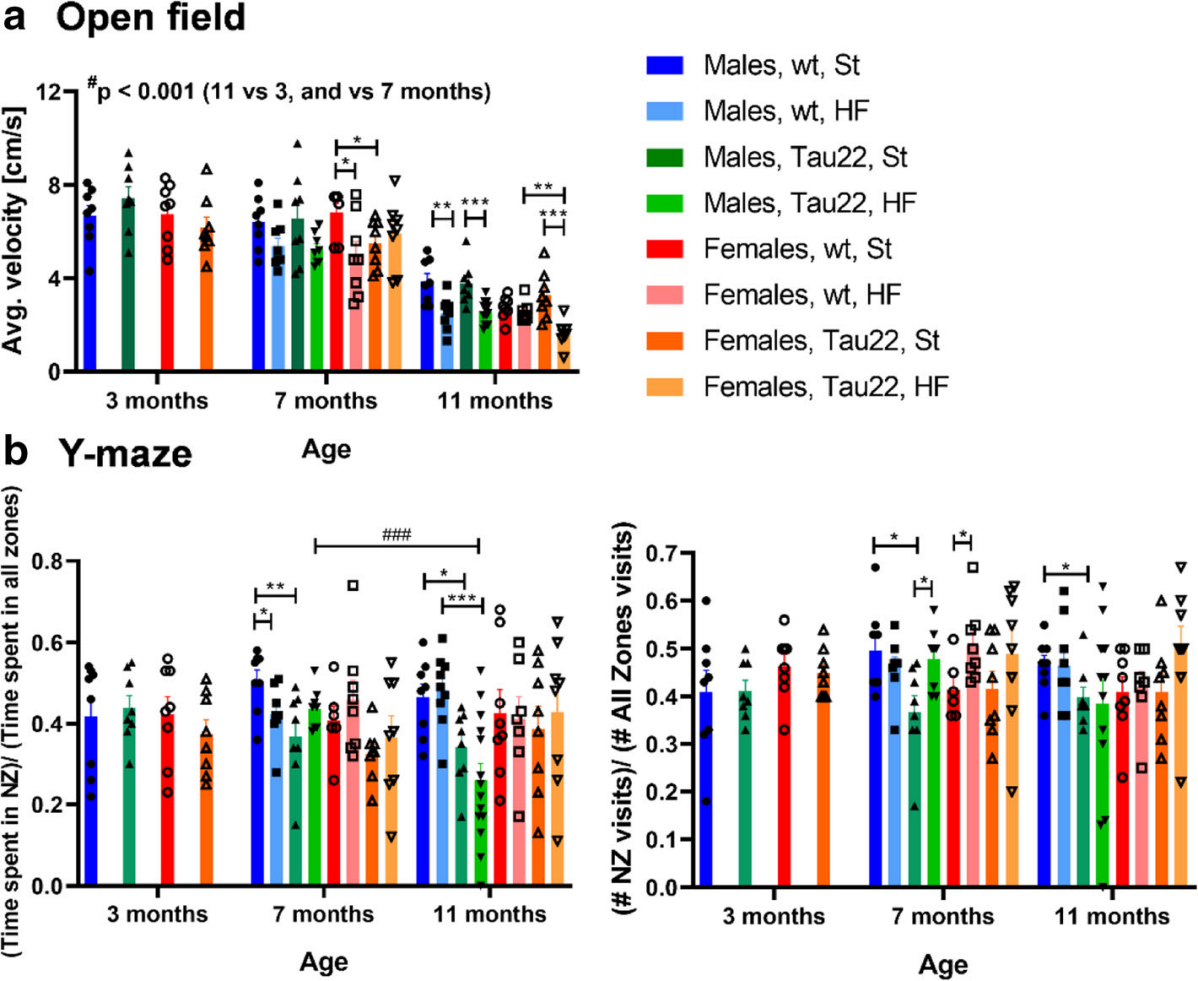
Behavioral tests. **a** Open field (n = 7–14). **b** Y-maze (n = 6–14). Data are presented as mean ± SEM and were statistically analyzed by Mann-Whitney t test (*) within each age and sex group (*p < 0.05; **p < 0.01). The age comparison was performed by mixed-effects analysis and Bonferroni’s post hoc test (^#^). A value significance between 11 and 3, and 11 and 7 months in a particular group at an average velocity of open field test was ^###^p < 0.001 in all cases (not shown for clarity). NZ, new zone

Age significantly decreased mobility, indicated by a decreased average velocity and running distance, in all older groups compared to the younger groups (Fig. 1A). The 11-month-old mice also showed a higher anxiety-like tendency, which was expressed by a shorter mean wall distance (Supplementary Fig. 1). Furthermore, the HF diet slightly or significantly decreased the mobility of all mouse groups, except for 7-month-old THY-Tau22 females and 11-month-old wt females. Out of all groups, the 11-month-old THY-Tau22 females fed a HF diet showed the lowest mobility. Interestingly, the HF diet caused a significant increase of anxiety-like behavior of THY-Tau22 females when compared to wt controls expressed by the lowest wall distance (Supplementary Fig. 1).

### The combination of age and THY-Tau22 genotype significantly worsened short-term spatial memory in males

Female groups showed no differences in the time spent in the NZ of the Y-maze (Fig. 1B). In contrast, 7- and 11-month-old THY-Tau22 males fed the St diet showed a poor willingness to spend time in the NZ when compared to wt controls (Fig. 1B). Furthermore, 11-month-old THY-Tau22 males on HF diet showed an even stronger decrease when compared to the same diet wt controls (Fig. 1B); nevertheless, the number of NZ visits for 11-month-old THY-Tau22 males did not show a significant decrease when compared to the same diet wt controls (Fig. 1B). The number of NZ visits significantly decreased in 7- and 11-month-old males on St diet when compared to wt controls (Fig. 1B).

### HF diet significantly increased the body weight and the fat weight of mice

**Fig. 2:**
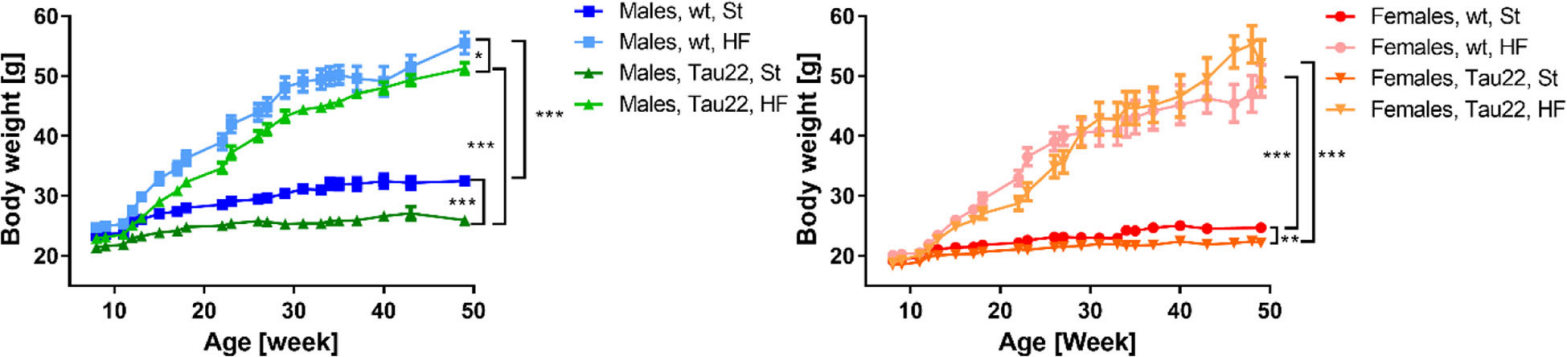
Body weight of THY-Tau22 and wt mice. Males: n = 8–23, females: 7–21. Data are presented as mean ± SEM. The comparison of final weights (males: n = 8–14, females: 7–8) was statistically analyzed by Mann-Whitney t test (*p < 0.05; **p < 0.01; ***p < 0.001)

The HF diet significantly increased the body weight of all mice (Fig. 2). The body weights of THY-Tau22 males and females fed the St diet were lower than those of their respective wt controls. The same trend was observed in males but not females fed the HF diet (Fig. 2).

**Fig. 3:**
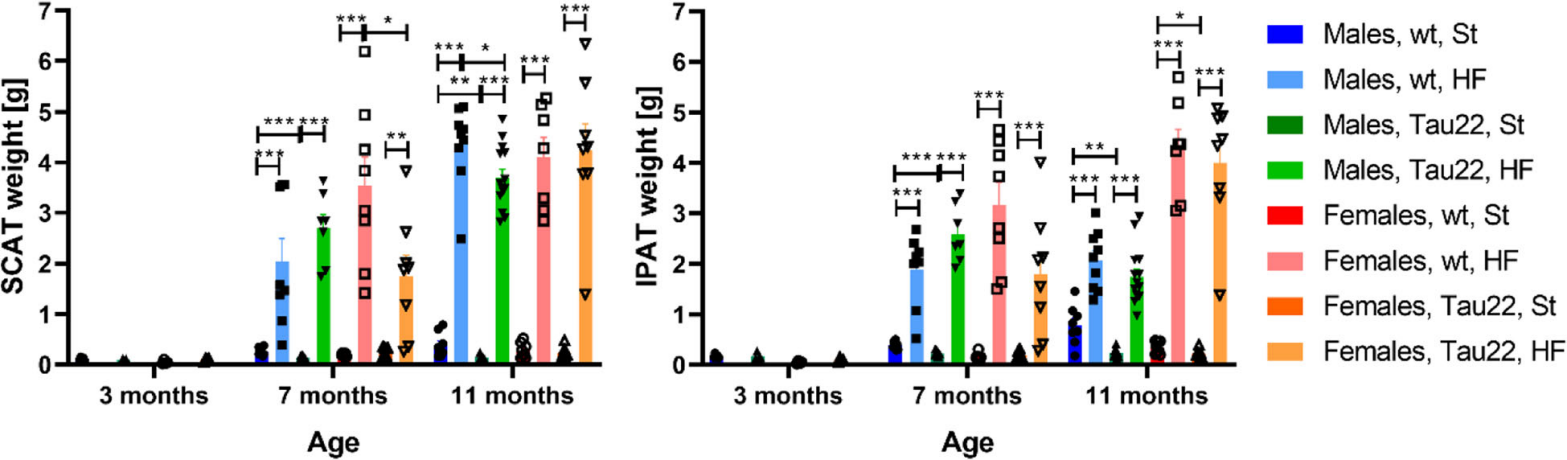
Adipose tissues weight of THY-Tau22 and wt mice. Subcutaneous (SCAT) and intraperitoneal (IPAT) adipose tissue weight of 3- (n = 4–5), 7- (n = 7–8), and 11-month-old (n = 7–14) mice. Data are presented as mean ± SEM and were statistically analyzed by Mann-Whitney t test within each age and sex group (*p < 0.05; **p < 0.01; ***p < 0.001)

Accordingly, the weights of SCAT and IPAT were significantly increased by the HF diet in 7- and 11-month-old mice of both sexes (Fig. 3). Furthermore, 7- and 11-month-old THY-Tau22 males on the St diet showed lower weights of SCAT and IPAT than their respective wt controls (Fig. 3). The weight of SCAT correlated with the weight of IPAT in 7- but not 11-month-old animals of both sexes (Fig. 3).

**Fig. 4:**
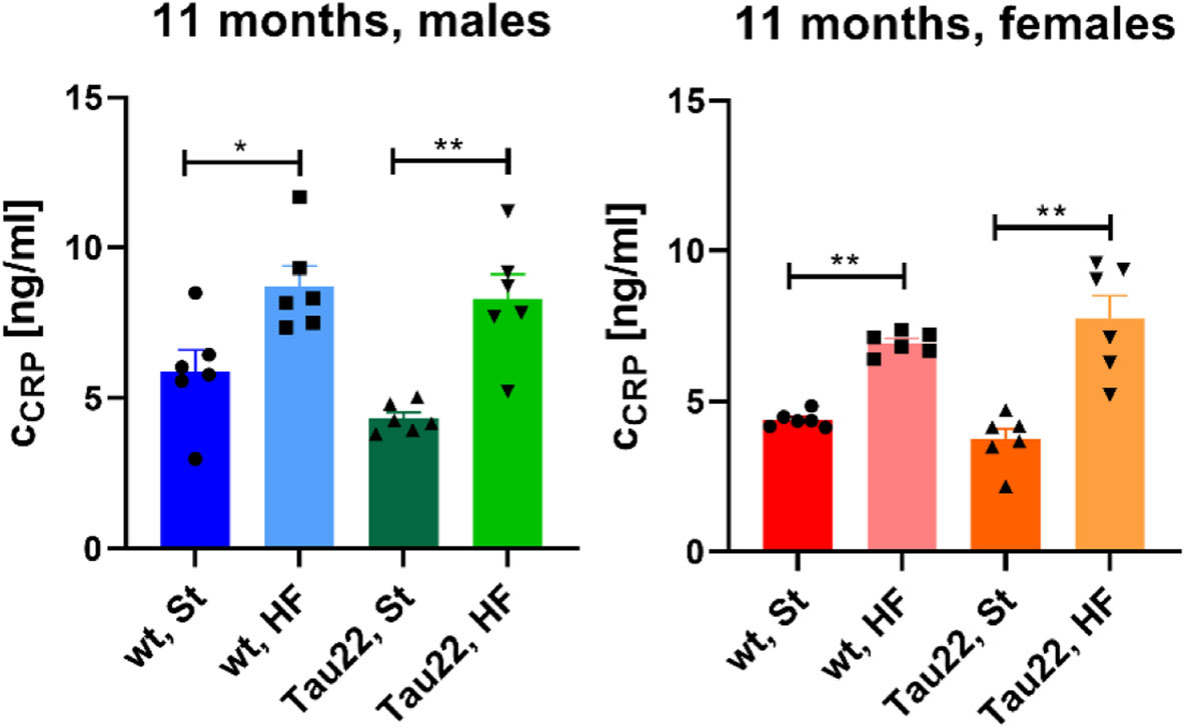
Inflammation marker in the blood plasma. The concentration of C-reactive protein (CRP) in non-anesthetized fasting plasma of 11-month-old male and female mice (n = 6). Data are presented as mean ± SEM and were statistically analyzed by Mann-Whitney t test within each age and sex group (*p < 0.05; **p < 0.01)

Furthermore, the HF diet-induced obesity led to a significant increase of C-reactive protein (CRP), the inflammation marker, in the fasting plasma of 11-month-old animals, when compared to the St diet-fed animals (Fig. 4).

### HF diet caused liver steatosis

**Fig. 5:**
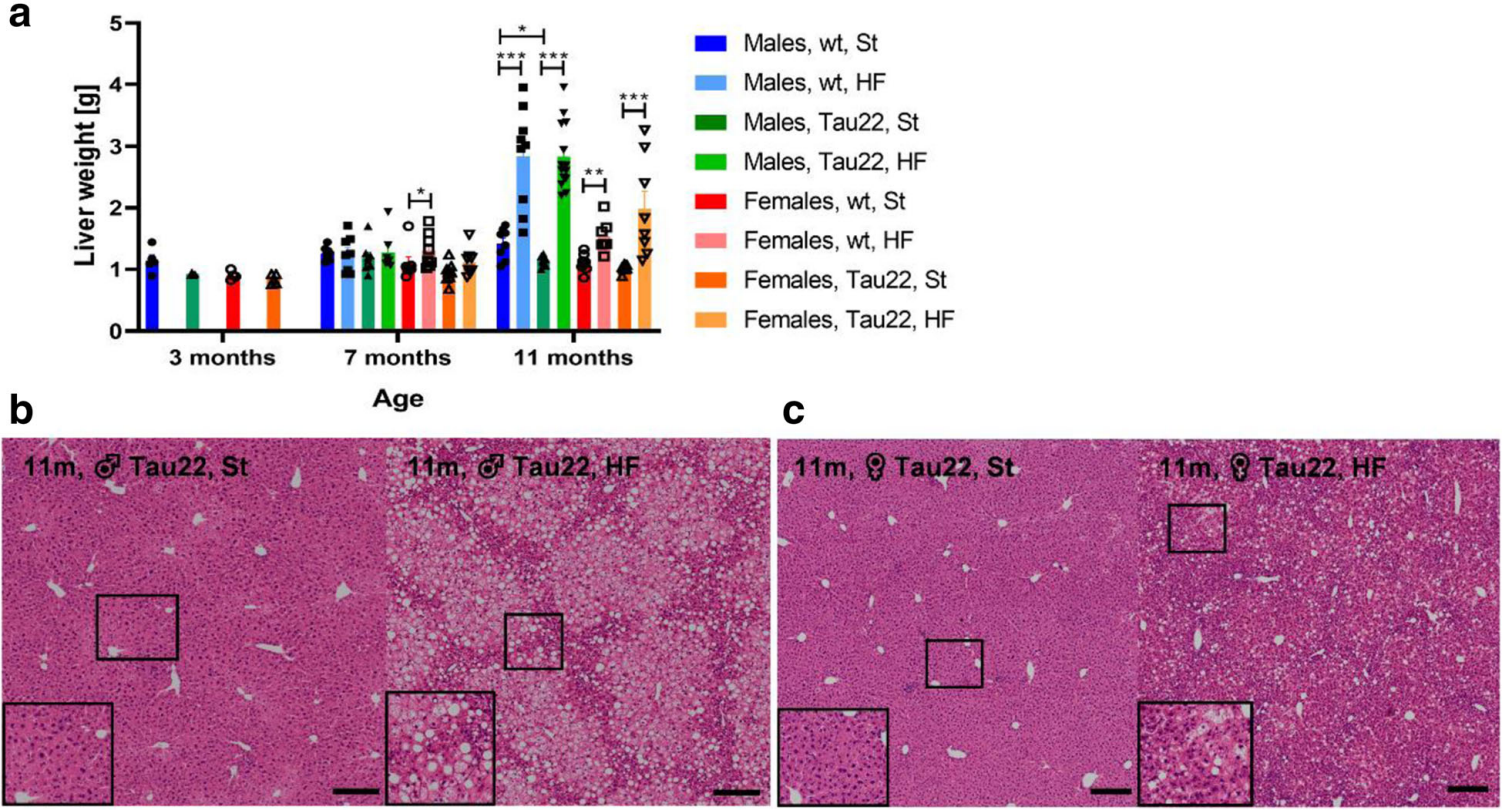
Liver weight and liver steatosis of THY-Tau22 and wt mice. **a** Liver weight at 3, 7, and 11 months of age (males: n = 4–14, females: n = 4–8). Data are presented as mean ± SEM and were statistically analyzed by Mann-Whitney t test within each age and sex group (*p < 0.05; **p < 0.01; ***p < 0.001). **b** A representative figure of liver hematoxylin-eosin (HE) stained 11-month-old THY-Tau22 males on St (left) and HF (right) diet. **C** A representative figure of HE stained 11-month-old THY-Tau22 females on St (left) and HF (right) diet. Black-framed inserts in left down corners show a magnified area in black frames. Scale bar 200 μm, ×20 magnification

HF diet feeding generally caused a significant increase in liver weight and typical liver steatosis in all 11-month-old mice (Fig. 5). Representative figures of liver slices from 11-month-old transgenic males and females on both diets are shown in Fig. 5B and C, respectively. In contrast, St diet-fed mice did not show any evidence of liver steatosis, even at a late age (Fig. 5).

### HF diet significantly increased plasma leptin and lipids

The HF diet increased the plasma level of leptin very substantially in all 7- and 11-month-old mice, in line with body weight and SCAT and IPAT weight (Tables 5, 6, and 7; Figs. 2 and 3). A similar trend was observed for plasma cholesterol, except for 7-month-old males (Tables 5, 6, and 7).

**Table 5:** Metabolic parameters of THY-Tau22 and wt mice. Three-month-old

Parameter	Sex	wt St	Tau22 St
Fasting glucose [mmol/l] (n = 4–5)	Male	6.32±0.30	5.93±0.36
Cholesterol [mmol/l] (n = 4–5)	Male	1.24±0.07	1.20±0.06
FFA [ng/ml] (n = 4–5)	Male	0.24±0.08	0.34±0.06
TG [mmol/l] (n = 4–5)	Male	0.22±0.05	0.30±0.04
Leptin [ng/ml] (n = 4–5)	Male	0.59±0.06	0.51±0.03
Fasting glucose [mmol/l] (n = 4–5)	Female	5.30±0.43	4.00±0.28^+^
Cholesterol [mmol/l] (n = 4–5)	Female	0.97±0.09	1.01±0.08
FFA [ng/ml] (n = 4–5)	Female	0.10±0.02	0.61±0.09^+^
TG [mmol/l] (n = 4–5)	Female	0.25±0.04	0.25±0.03
Leptin [ng/ml] (n = 4–5)	Female	0.52±0.08	0.87±0.18

Fasting glucose, cholesterol, triglycerides (TG), free fatty acids (FFA), and leptin were measured in 3-, 7-, and 11-month-old males and females of both diets. The data are presented as mean ± SEM and were statistically analyzed by Mann-Whitney t test within each age and sex group (*HF vs St within the wt or Tau22 group, ^+^Tau22 vs wt on the same diet). The age comparison was performed using mixed-effects analysis and Bonferroni’s post hoc test (^#^7 or 11 vs 3 months, ^$^11 vs 7 months). Statistical significance for all measurements: *p < 0.05; **p < 0.01; ***p < 0.001

**Table 6:** Metabolic parameters of THY-Tau22 and wt mice. Seven-month-old

Parameter	Sex	wt St	wt HF	Tau22 St	Tau22 HF
Fasting glucose [mmol/l] (n = 7–8)	Male	8.98±0.43^#^	10.80±0.80*	9.39±1.03^#^	10.73±0.72
Cholesterol [mmol/l] (n = 7–8)	Male	1.06±0.16	1.64±0.26	0.88±0.05	1.87±0.13***
FFA [ng/ml] (n = 7–8)	Male	0.39±0.05	0.48±0.07	0.45±0.09	0.77±0.07*^, +^
TG [mmol/l] (n = 7–8)	Male	0.48±0.11^#^	0.63±0.12	0.36±0.03	0.78±0.08***
Leptin [ng/ml] (n = 7–8)	Male	3.22±0.49	70.76±19.41**	1.68±0.60	61.82±12.30***
Fasting glucose [mmol/l] (n = 7–8)	Female	7.47±0.84	9.61±0.38*	8.10±0.97^###^	10.19±0.66
Cholesterol [mmol/l] (n = 7–8)	Female	0.57±0.03^##^	1.23±0.08***	0.62±0.05^###^	1.33±0.13***
FFA [ng/ml] (n = 7–8)	Female	0.40±0.08^#^	0.46±0.03	0.52±0.08	0.56±0.04
TG [mmol/l] (n = 7–8)	Female	0.31±0.03	0.46±0.03**	0.58±0.28	0.40±0.02
Leptin [ng/ml] (n = 7–8)	Female	3.19±0.80^#^	92.22±14.35***	2.95±0.27	42.06±12.20***^, +^

Fasting glucose, cholesterol, triglycerides (TG), free fatty acids (FFA), and leptin were measured in 3-, 7-, and 11-month-old males and females of both diets. The data are presented as mean ± SEM and were statistically analyzed by Mann-Whitney t test within each age and sex group (*HF vs St within the wt or Tau22 group, ^+^Tau22 vs wt on the same diet). The age comparison was performed using mixed-effects analysis and Bonferroni’s post hoc test (^#^7 or 11 vs 3 months, ^$^11 vs 7 months). Statistical significance for all measurements: *p < 0.05; **p < 0.01; ***p < 0.001

**Table 7:** Metabolic parameters of THY-Tau22 and wt mice. Eleven-month-old

Parameter	Sex	wt St	wt HF	Tau22 St	Tau22 HF
Fasting glucose [mmol/l] (n = 8–14)	Male	7.74±0.69	9.06±0.63	7.11±0.58	8.74±0.26*
Cholesterol [mmol/l] (n = 7–14)	Male	0.98±0.07	3.25±0.17***^, $$$^	1.00±0.05	3.14±0.18***^, $$$^
FFA [ng/ml] (n = 8–13)	Male	0.50±0.13	0.67±0.06	0.48±0.09	0.83±0.06**
TG [mmol/l] (n = 7–14)	Male	0.35±0.03	0.53±0.04*	0.27±0.04	0.49±0.04***^, $$^
Leptin [ng/ml] (n = 5–14)	Male	4.71±2.06^#^	42.59±1.63***	0.58±0.28^+^	47.47±1.05***^, ++^
Fasting glucose [mmol/l] (n = 7–8)	Female	7.70±0.42	6.13±0.30*^, $$$^	7.76±0.34^##^	7.88±0.55^+, $^
Cholesterol [mmol/l] (n = 7–8)	Female	0.72±0.05	1.92±0.13***^, $$$^	0.76±0.09^#^	2.04±0.16***^, $$$^
FFA [ng/ml] (n = 6–8)	Female	0.41±0.04^#^	0.68±0.07**^, $^	0.34±0.04^#, $^	0.57±0.04**
TG [mmol/l] (n = 7–8)	Female	0.38±0.05	0.56±0.05**	0.39±0.04	0.39±0.04^+^
Leptin [ng/ml] (n=7–8)	Female	4.63±0.82^##^	38.40±1.65***^, $$$^	3.36±0.76 ^#^	42.09±3.92***

Fasting glucose, cholesterol, triglycerides (TG), free fatty acids (FFA), and leptin were measured in 3-, 7-, and 11-month-old males and females of both diets. The data are presented as mean ± SEM and were statistically analyzed by Mann-Whitney t test within each age and sex group (*HF vs St within the wt or Tau22 group, ^+^Tau22 vs wt on the same diet). The age comparison was performed using mixed-effects analysis and Bonferroni’s post hoc test (^#^7 or 11 vs 3 months, ^$^11 vs 7 months). Statistical significance for all measurements: *p < 0.05; **p < 0.01; ***p < 0.001

Unexpectedly, the THY-Tau22 genotype caused a significant increase in FFA in 3-month-old females. The HF diet enhanced plasma FFA in all 11-month-old but not in 7-month-old mice, where no difference in FFA among groups was observed. TG levels were increased ambiguously in some groups (Tables 5, 6, and 7).

### No significant differences among groups were detected in tolerance to glucose

Fasting glucose levels were normal at 3 months of age in all groups. At 7 months, the fasting glucose level increased nondramatically above the normoglycemic value (the value for lean wt mice), but at 11 months of age, it did not rise again. HF-diet feeding increased fasting glucose only in 7-month-old wt males and females (Tables 5, 6, and 7).

**Fig. 6:**
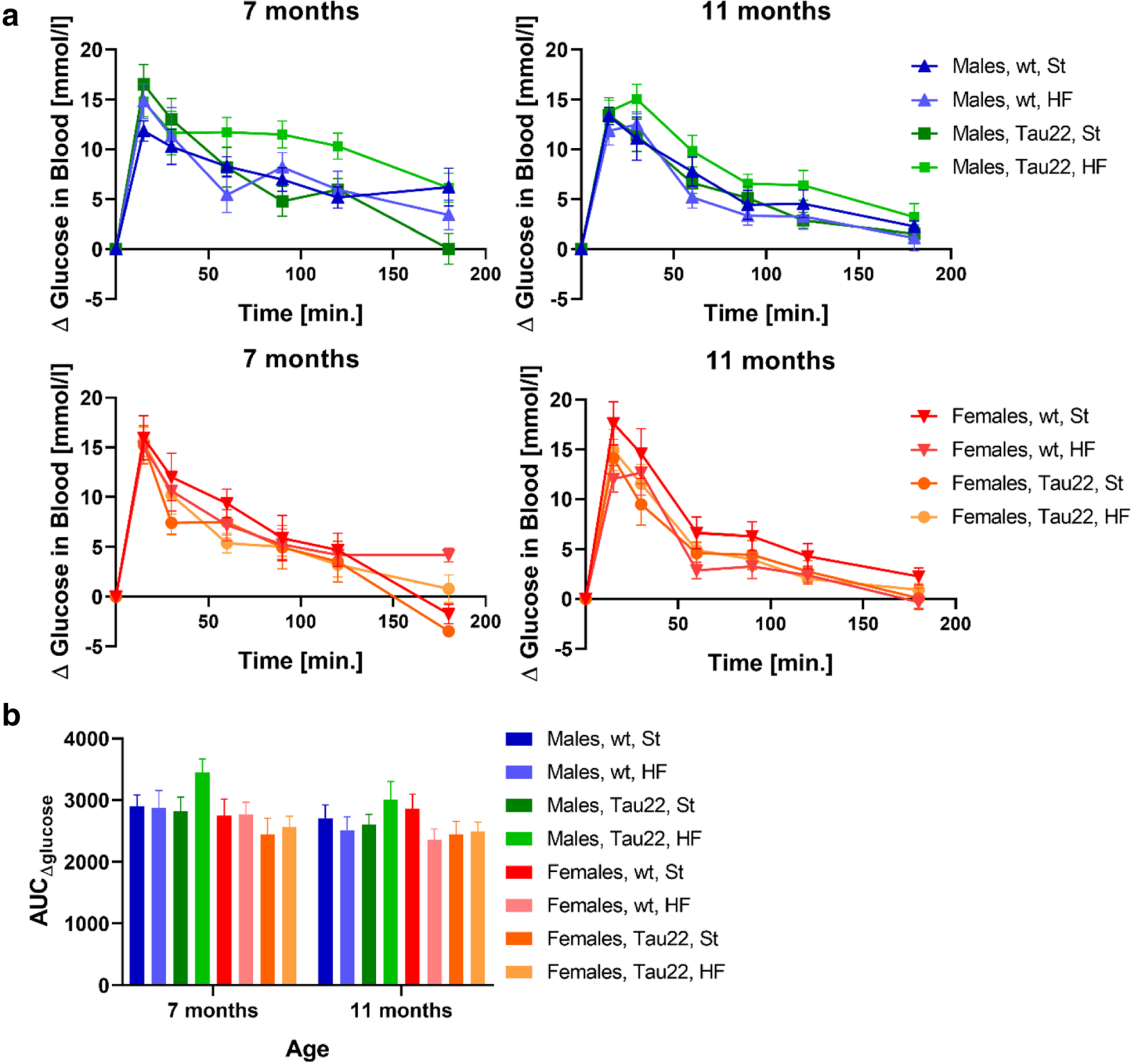
Oral glucose tolerance test (OGTT) in THY-Tau22 and wt mice. **a** OGTT results are shown as ΔGlucose profile in 7- and 11-months-old mice (n = 7–8 and 7–14, respectively). **b** Graph corresponds to the calculated areas under the curves (AUC_Δglucose_) from the **a** graphs. The data in **b** graph are presented as mean ± SEM and were statistically analyzed using non-parametric unpaired Kruskal-Wallis one-way ANOVA and Bonferroni’s post hoc test within each age and sex group. The age comparison was performed by mixed-effects analysis and Bonferroni’s post hoc test. No statistically significant difference was observed (p ≥ 0.05)

The OGTT AUC values did not show any significant differences among the tested groups, both 7- and 11-month-old mice (Fig. 6).

### HF diet-fed mice showed increased peripheral insulin resistance

The HF diet significantly increased the concentration of fasting plasma insulin in all groups (Table 8). Even though fasting glucose was increased only slightly by the HF diet, all mice fed the HF diet showed a significantly higher HOMA-IR index than their respective St diet-fed controls (Table 8), which indicates increased peripheral IR. HOMA-IR indexes also increased in 11-month-old wt males on the St diet and THY-Tau22 females on the HF diet compared to the respective 7-month-old controls (Table 8).

**Table 8:** Insulin concentration and HOMA-IR and QUICKI indexes as insulin resistance quantification of THY-Tau22 and wt mice

	Sex	Age [months]	wt St	wt HF	Tau22 St	Tau22 HF
Fasting insulin [ng/ml]	Male (n = 7–8)	7	0.18±0.02	1.07±0.43**	0.15±0.03	1.17±0.32***
	Male (n = 7–12)	11	0.40±0.11^$^	7.80±3.96**^, $^	0.15±0.05	2.43±0.85***
	Female (n = 7–8)	7	0.17±0.03	0.57±0.14**	0.12±0.03	0.35±0.06**
	Female (n = 6–7)	11	0.15±0.07	1.87±0.82**	0.22±0.06	3.58±1.57**^, $$^
HOMA-IR	Male (n = 7–8)	7	12.45±1.65	89.43±36.83***	10.81±2.83	102.47±32.20**
	Male (n = 7–12)	11	23.66±6.00	475.67±210.86**^, $^	8.53±2.85	150.95±47.97***
	Female (n = 7–8)	7	10.22±2.37	41.69±9.68**	7.36±1.86	26.52±4.33**
	Female (n = 6–7)	11	9.08±4.12	83.87±32.06**	14.16±4.31	229.88±106.68**^, $$^
QUICKI	Male (n = 7–8)	7	0.35±0.01	0.29±0.01***	0.38±0.02	0.28±0.01**
	Male (n = 7–12)	11	0.35±0.02	0.25±0.01**	0.39±0.01	0.27±0.01***
	Female (n = 7–8)	7	0.37±0.01	0.31±0.01**	0.40±0.02	0.32±0.01**
	Female (n = 6–7)	11	0.55±0.12^$^	0.29±0.01**	0.36±0.02	0.25±0.02**

Fasting insulin was measured in 7- and 11-month-old males and females of both diets. HOMA-IR and QUICKI indexes [23, 24] were calculated from the fasting insulin and fasting glucose concentrations (Table 5) for 7- and 11-month-old mouse groups. The data are presented as mean ± SEM and were statistically analyzed by Mann-Whitney t test within each age and sex group (*HF vs St within the wt or Tau22 group). The age comparison was performed using mixed-effects analysis and Bonferroni’s post hoc test (^$^11 vs 7 months). Statistical significance for all measurements: *p < 0.05; **p < 0.01; ***p < 0.001. *HOMA-IR* homeostatic model assessment for insulin resistance, *QUICKI* quantitative insulin sensitivity check index

The HF diet also caused a significant decrease in the QUICKI index in all mice. There was a significant increase in the QUICKI index between 7 and 11 months in wt females on the St diet (Table 8).

### THY-Tau22 genotype and HF diet significantly increased neuroinflammation

Neuroinflammation is typically characterized by a higher number of clusters of microglia (marked by Iba1) and activated astrocytes (marked by GFAP).

**Fig. 7:**
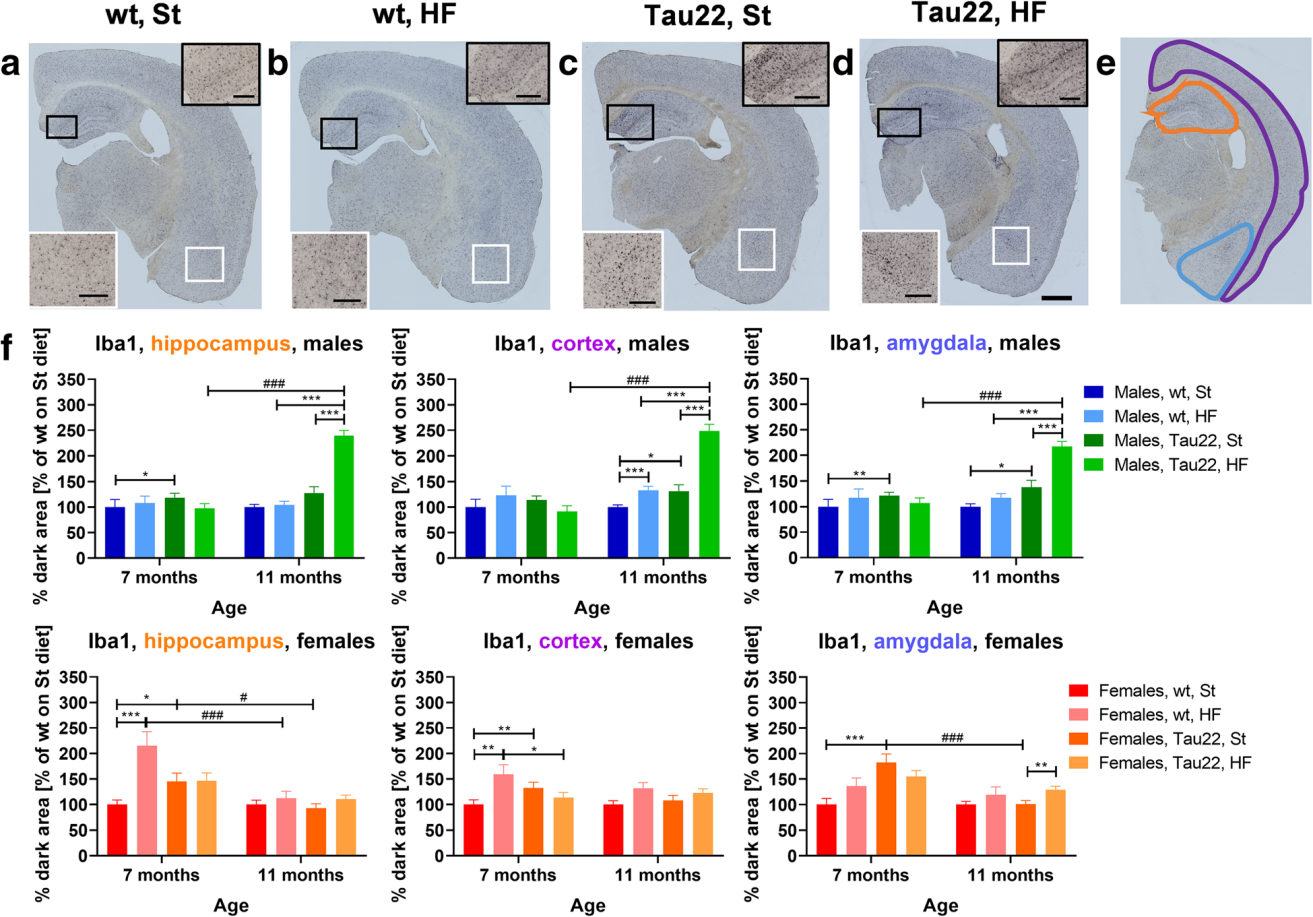
Microgliosis marker. Iba1 DAB-stained representative right hemisphere brain sections of 11-month-old males **a** wt on St diet, **b** wt on HF diet, **c** THY-Tau22 on St diet, **d** THY-Tau22 on HF diet. Black- and white-framed inserts in left down and up right corners show magnified areas in particular color frames. Scale bar for the whole section 500 μm, for inserts 200 μm. **e** A representative figure of analyzed areas: hippocampus (orange), cortex (purple), and amygdala (blue). **f** Relative quantification at particular analyzed area (**e**) at right hemisphere brain sections of 7- and 11-month-old THY-Tau22 and wt mice (n = 20–25 sections per 4 mice). Wt group on St diet was set as 100 %. Data are presented as mean ± SEM and were statistically analyzed using Mann-Whitney t test within each age and sex group (*). The age comparison of brain sections (**f**) was performed by mixed-effects analysis and Bonferroni’s post hoc test (^#^). Statistical significance for all measurements: *p < 0.05; **p < 0.01; ***p < 0.001. *DAB* 3′,3′-diaminobenzidine, *Iba1* ionized calcium-binding adapter molecule 1

Iba1 differed among groups of the same age in males and females. Among the 7-month-old males, only the THY-Tau22 males on the St diet showed increased Iba1 levels in the hippocampus and amygdala compared to wt controls. In females of the same age, Iba1 was increased significantly in the amygdala in THY-Tau22 females on the St diet compared to wt females. Furthermore, the HF diet increased Iba1 in the hippocampus and cortex of 7-month-old wt females. However, in 11-month-old females, nearly no difference in Iba1 was registered among groups in all three areas. On the other hand, 11-month-old THY-Tau22 males fed the HF diet showed massively increased Iba1 in all three areas compared to THY-Tau22 males fed the St diet or wt HF diet. In addition, Iba1 was significantly increased in 11-month-old THY-Tau22 males fed the HF diet compared to those 7-month-old fed the HF diet (Fig. 7, Supplementary Fig. 2). WB performed in the hippocampus labeled with anti-CD11b antibody showed only one significant increase in 7-month-old transgenic females fed the HF diet when compared to wt females fed the same diet (Supplementary Fig. 2).

**Fig. 8:**
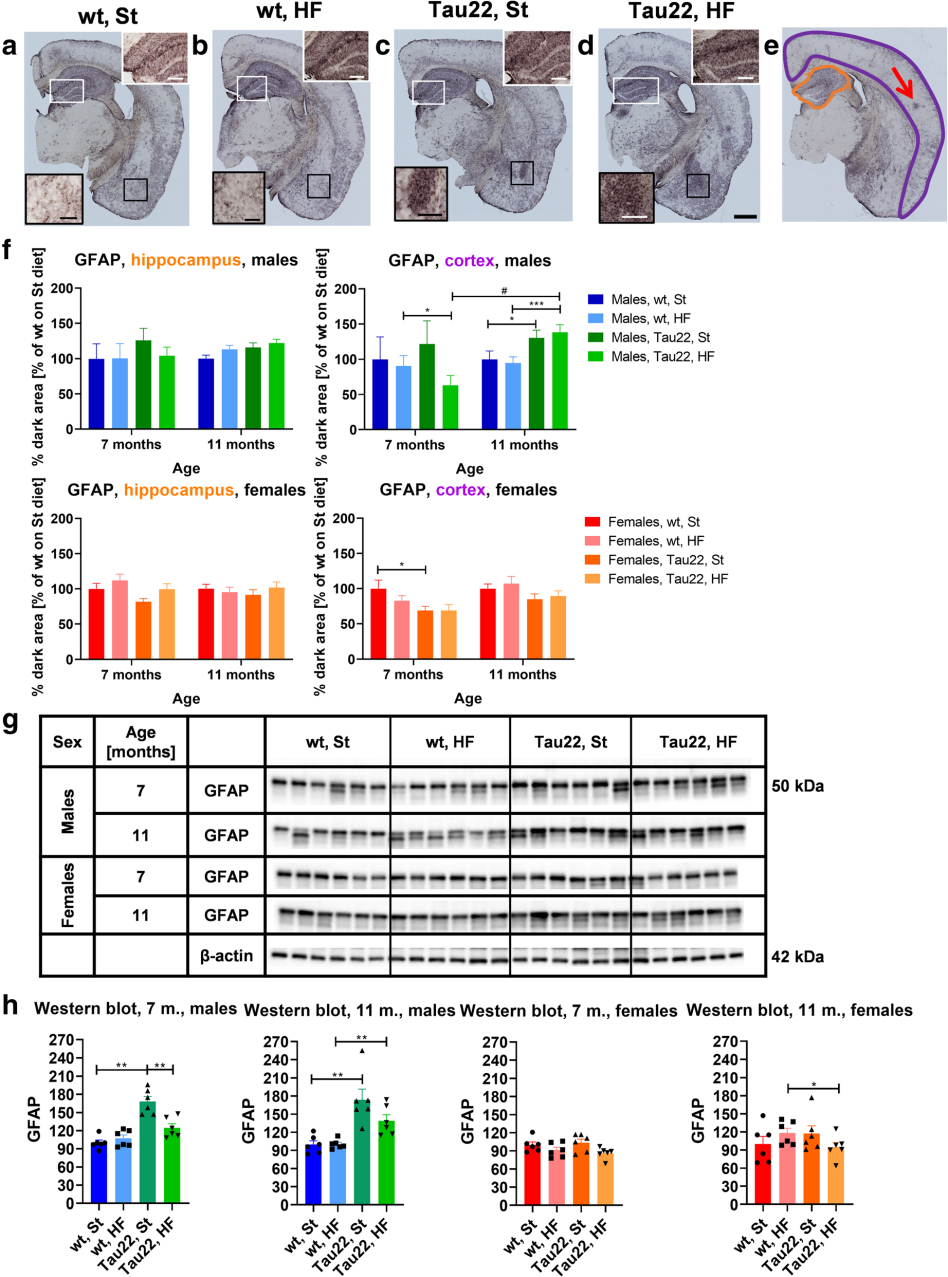
Astrocytosis marker. GFAP DAB-stained representative right hemisphere brain sections of 11-month-old males **a** wt on St diet, **b** wt on HF diet, **c** THY-Tau22 on St diet, **d** THY-Tau22 on HF diet. Black- and white-framed inserts in left down and up right corners show magnified area in particular color frames. Scale bar for the whole section 500 μm, for inserts 200 μm. **e** A representative figure of analyzed areas: hippocampus (orange), cortex (purple). The red arrow point to cluster of reactive astrocytes. **f** Relative quantification at particular analyzed area (**e**) at right hemisphere brain sections of 7- and 11-month-old THY-Tau22 and wt mice (n = 24 sections per 4 mice). Mouse wt group on St diet was set as 100 %. **g** Western blots of hippocampal GFAP. **h** Quantification of **g** western blots of hippocampal GFAP in 7- and 11-month-old mice (n = 6). Mouse wt group on St diet was set as 100 %. The intensity of GFAP was related to particular β-actin intensity. All data are presented as mean ± SEM and were statistically analyzed using Mann-Whitney t test within each age and sex group (*). The age comparison of brain sections (F) was performed by mixed-effects analysis and Bonferroni’s post hoc test (^#^). Statistical significance for all measurements: *p < 0.05; **p < 0.01; ***p < 0.001. *DAB* 3′,3′-diaminobenzidine, *GFAP* glial fibrillary acidic protein

Seven-month-old males did not show any significant difference in hippocampal GFAP detected by IHC, as did females (Fig. 8F). In contrast, the WB of hippocampal GFAP showed increased GFAP in 7-month-old THY-Tau22 males on the St diet and 11-month-old THY-Tau22 on both diets compared to wt males, but this effect was not observed in females (Fig. 8G and H). On the other hand, IHC results of 11-month-old THY-Tau22 males on both diets showed increased GFAP in the cortex compared to the wt males (Fig. 8F).

**Fig. 9:**
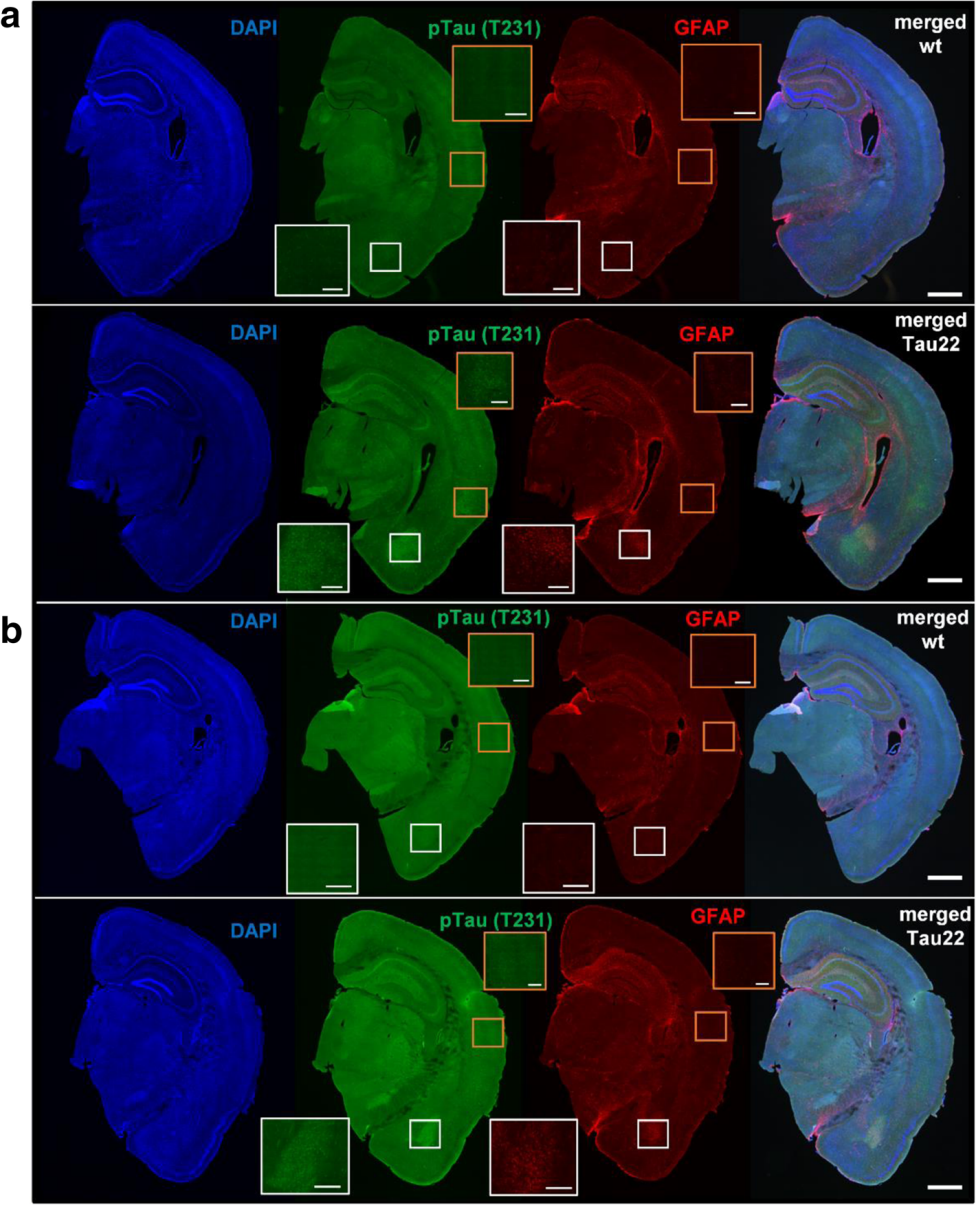
Fluorescence-labeled THY-Tau22 and wt mouse brains. Colocalization of cell nuclei (blue DAPI), pTau (T231) (green AT180), and activated astrocytes (red GFAP). The representative figures of right hemisphere brain sections of **a** 7-month-old wt male mouse (upper pictures series) and THY-Tau22 mouse (lower pictures series), both on St diet, and **b** 11-month-old wt male mouse (upper pictures series) and THY-Tau22 male mouse (lower pictures series), both on HF diet, stained with particular fluorescence antibodies. White- and orange-framed inserts in left down and up right corners show magnified areas in particular color frames. Scale bar for whole brain section 500 μm, for inserts 200 μm. *GFAP* glial fibrillary acidic protein, *DAPI* 4′,6-diamidin-2-fenylindol

Finally, fluorescent staining was used for colocalization of activated astrocytes, pTau (T231), and neuron nuclei. The representative figures illustrate 7-month-old THY-Tau22 males and their wt controls, both on the St diet (Fig. 9A), and 11-month-old THY-Tau22 males and their wt controls, both on the HF diet (Fig. 9B). As shown in Fig. 9, pTau (T231) was abundantly present in the brains of THY-Tau22 males of both ages. GFAP was observed in close vicinity to pTau (T231), mostly in the amygdala and hippocampus. The abundance of pTau (T231) was also observed in the cortex of THY-Tau22 males (Fig. 9).

### The combination of the THY-Tau22 genotype and HF diet significantly worsened hippocampal synaptic plasticity in 11-month-old female mice

**Fig. 10:**
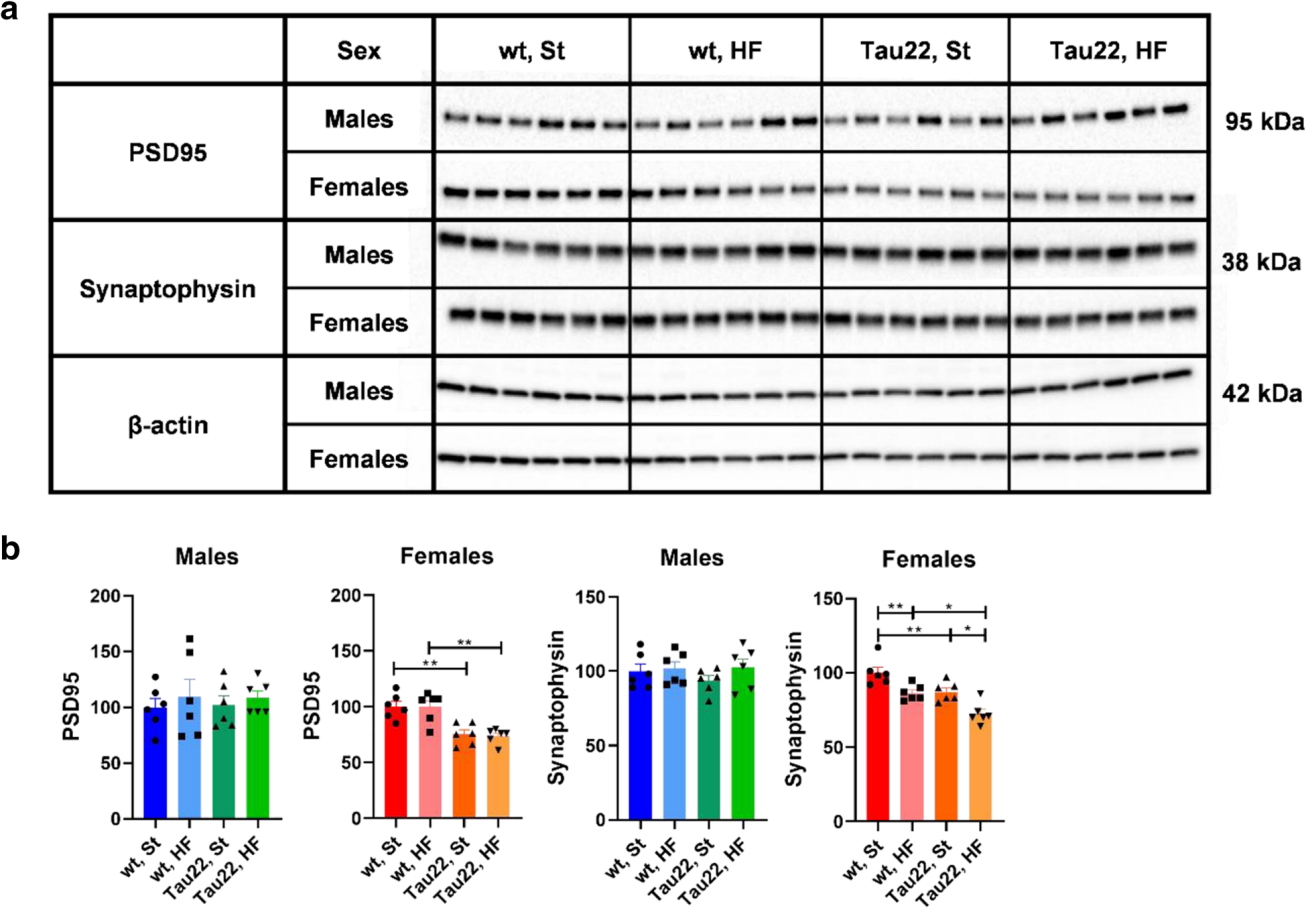
Western blots of hippocampal synaptic plasticity markers of 11-month-old THY-Tau22 and wt mice. **a** Western blots for specific proteins. **B** Quantification of (**a**) western blots. Data are presented as mean ± SEM and were statistically analyzed by Mann-Whitney t test within each age and sex group (*p < 0.05; **p < 0.01; n = 6). Mouse wt group on St diet was set as 100%. The intensity of all proteins was related to particular β-actin intensity. PSD95: postsynaptic density protein 95

Synaptophysin and postsynaptic density protein 95 (PSD95) are markers of synaptic and postsynaptic plasticity, respectively. A significant decrease in the relative intensity of PSD95 and synaptophysin was shown in the hippocampi of 11-month-old transgenic females on both diets compared to their respective wt controls (Fig. 10). Furthermore, the HF diet itself significantly decreased synaptophysin in both THY-Tau22 and wt females. Interestingly, males of the same age did not show any significant difference in synaptic plasticity in the hippocampus among the groups (Fig. 10).

### The THY-Tau22 genotype and/or HF diet can differentially affect hippocampal insulin signaling in male and female mice

**Fig. 11:**
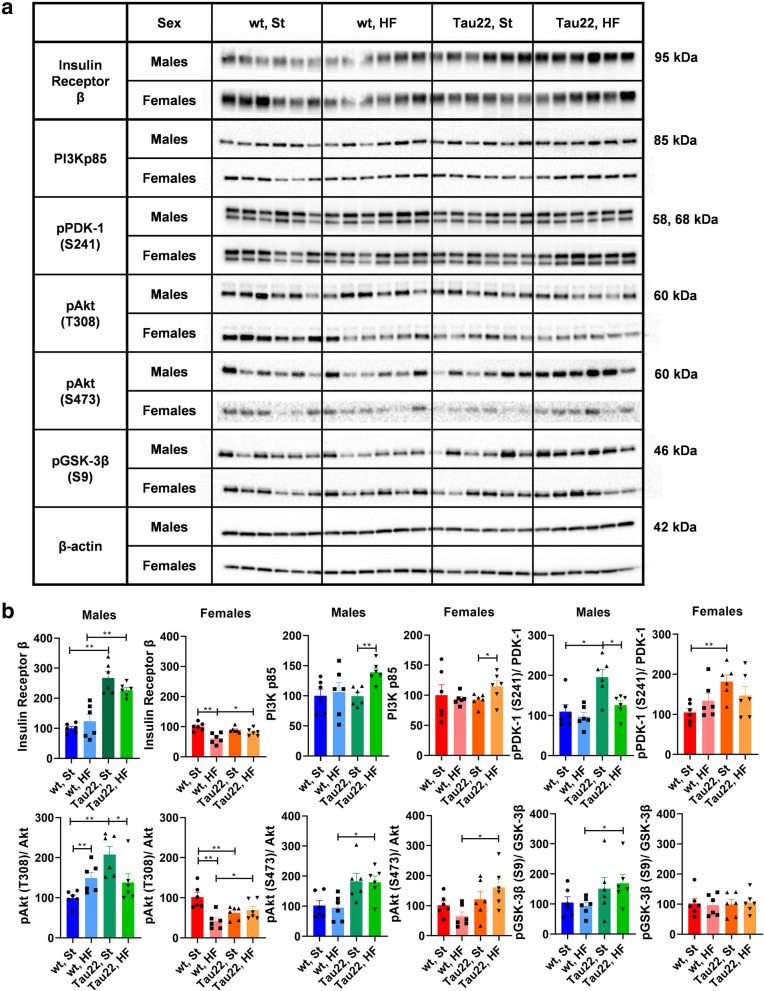
Western blots of hippocampal insulin signaling pathway markers of 11-month-old THY-Tau22 and wt mice. **a** Western blots for specific proteins. **b** Quantification of (**a**) western blots. Data are presented as mean ± SEM and were statistically analyzed by Mann-Whitney t test within each age and sex group (*p < 0.05; **p < 0.01; n = 6). Mouse wt group on St diet was set as 100 %. The intensity of all proteins was related to particular β-actin intensity. The intensity of phosphorylated protein was related to the total protein. pAkt, phosphorylated protein kinase B; pPDK-1 (S241), phosphoinositide-dependent kinase-1 phosphorylated at S241 residue; pGSK-3β, glycogen synthase kinase 3β phosphorylated at S9 residue; PI3Kp85, p85 regulatory subunit of phosphoinositide 3-kinase

Hippocampal Insulin Receptor β (IRβ) increased significantly in 11-month-old THY-Tau22 males fed both diets when compared to the corresponding controls. Interestingly, the HF diet-fed wt females showed lower hippocampal expression of IRβ, although this increased and reached a similar level to the rest of the female groups in THY-Tau22 HF diet-fed females (Fig. 11). Although the HF diet significantly increased the p85 regulatory subunit of phosphoinositide 3-kinase (PI3Kp85) in 11-month-old THY-Tau22 males and females, phosphoinositide-dependent kinase-1 phosphorylation at the S241 residue (pPDK-1 (S241)) was decreased in THY-Tau22 males fed the HF diet. THY-Tau22 of both sexes on the St diet had higher pPDK-1 than the respective wt controls (Fig. 11, Supplementary Fig. 3).

The phosphorylation of protein kinase B (Akt) at the T308 residue is mediated by PDK-1. Consistent with the hippocampal pPDK-1 results, increased phosphorylated pAkt (T308) was observed in 11-month-old THY-Tau22 males, but the HF diet decreased pAkt (T308) in THY-Tau22 males (Fig. 11, Supplementary Fig. 3). The THY-Tau22 genotype led to a significant increase in phosphorylated Akt at residue S473 (pAkt (S473)) in males and females fed the HF diet (Fig. 11, Supplementary Fig. 3). However, inhibitory pGSK-3β (S9) was increased, and thus, its kinase activity was attenuated in transgenic males fed the HF diet compared to the wt controls (Fig. 11, Supplementary Fig. 3).

### Tau phosphorylation in THY-Tau22 mice is independent of ERK1/2 and PP2A activity

**Fig. 12:**
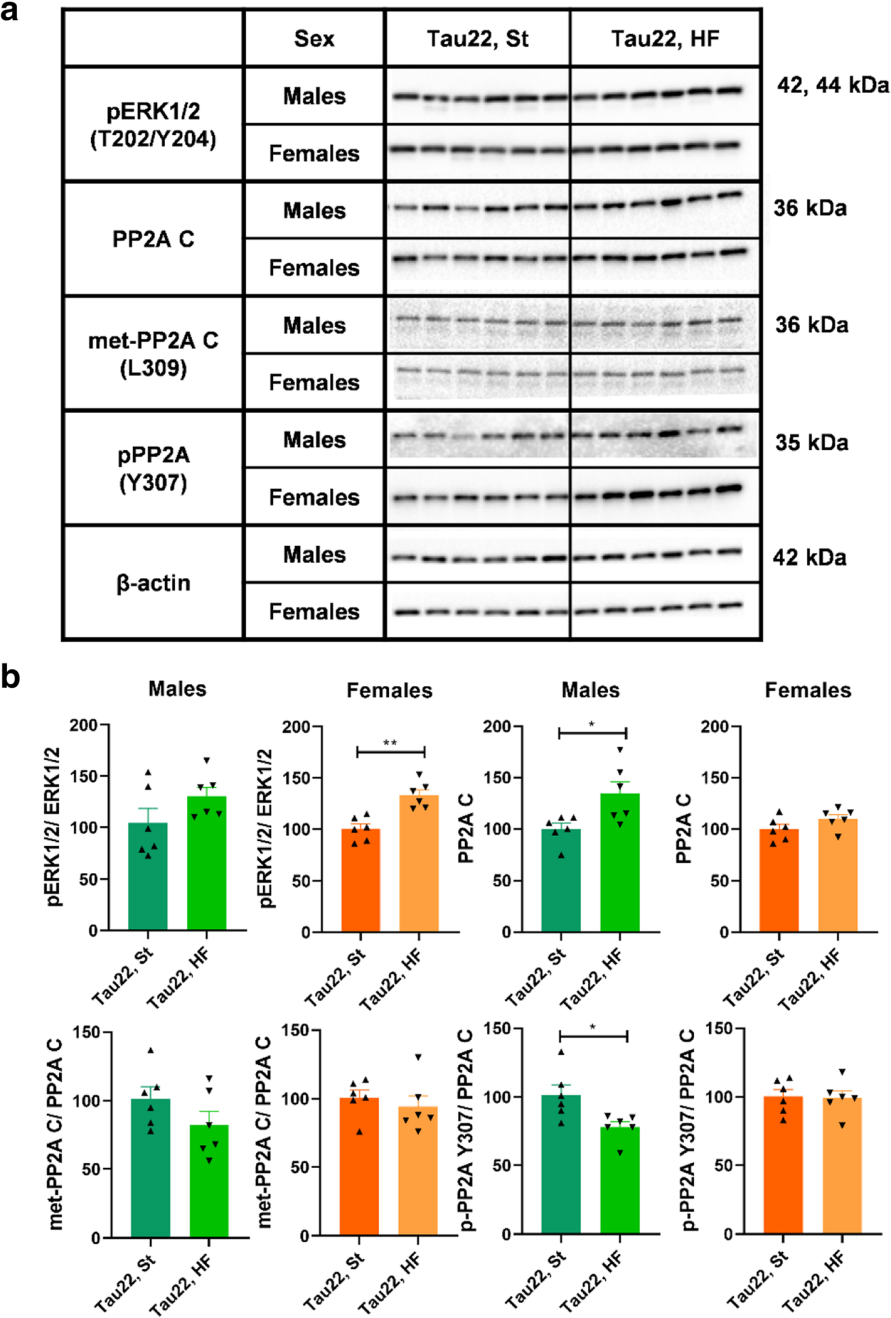
Western blots of hippocampal tau protein de/phosphorylation enzymes of 11-month-old THY-Tau22 mice. **a** Western blots for specific proteins. **b** Quantification of (**a**) western blots. Data are presented as mean ± SEM and were statistically analyzed by Mann-Whitney t test within each age and sex group (*p < 0.05; **p < 0.01; n = 6). Mouse THY-Tau22 group on St diet was set as 100 %. The intensity of all proteins was related to particular β-actin intensity. The intensity of modified protein was related to the total protein. pERK1/2, extracellular signal-regulated kinase phosphorylated at T202/Y204 residues; PP2A C, C subunit of protein phosphatase 2A; met-PP2A C (L309), methylated C subunit of PP2A at L309 residue; pPP2A C (Y307), PP2A C phosphorylated at Y307 residue

The HF diet significantly increased hippocampal extracellular signal-regulated kinase 1/2 phosphorylation at T202/Y204 residues (pERK1/2 (T202/Y204)) in THY-Tau22 females but not males (Fig. 12, Supplementary Fig. 4).

Hippocampal PP2A C was increased by HF diet feeding in THY-Tau22 males but not in females. The HF diet decreased the inactivated form of PP2A C, i. e. pPP2A C (Y307), in THY-Tau22 males. No further impact of the HF diet on the activity of PP2A was observed (Fig. 12).

Table 9 shows an overview of kinases and PP2A phosphatase used in this study, which have been found to directly de/phosphorylate tau protein at particular residues relevant for this study.

**Table 9:** Direct activity of kinases/ PP2A phosphatase on AD-potentially de/phosphorylated tau residues relevant for this study [25–29]

Phosphorylated tau residue	ERK1/2	GSK-3β	PP2A
**T212**	✓	✓	✓
**S214**		✓	✓
**T231**	✓	✓	
**S396**	✓	✓	✓
**S404**	✓	✓	✓
**S422**	✓		

*ERK1/2* extracellular signal-regulated kinase 1/2 known as mitogen-activated protein kinase MAPK, *GSK-3β* glycogen synthase kinase 3 beta; PP2A: protein phosphatase 2A

### The HF diet did not increase the relative intensity of phosphorylated tau proteins in transgenic mice

**Fig. 13:**
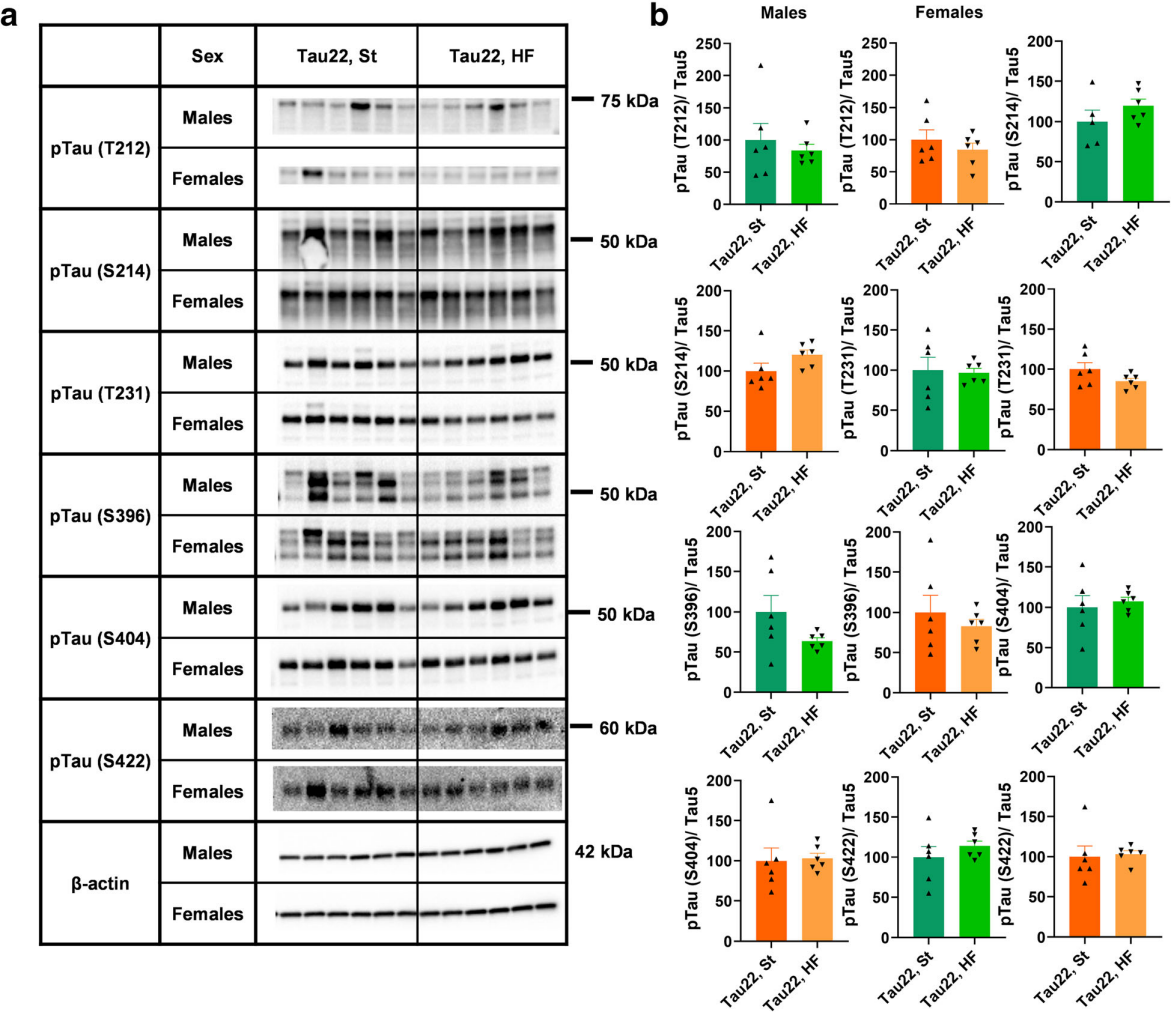
Western blots of hippocampal phosphorylated tau proteins of 11-month-old THY-Tau22 mice. **a** Western blots for specific proteins. **b** Quantification of (**a**) western blots. Data are presented as mean ± SEM and were statistically analyzed using Mann-Whitney t test within each age and sex group. No statistically significant difference was observed. (p ≥ 0.05; n = 5–6). Mouse THY-Tau22 group on St diet was set as 100%. The intensity of phosphorylated tau proteins was related to the Tau5 total protein

The impact of the HF diet on the phosphorylated tau proteins, pTau (phosphorylated at T212, S214, T231, S396, S404, and S422) related to Tau5 (total tau) in 11-month-old THY-Tau22 mice was determined. The results did not show any significant change in the relative pTau intensity for any epitope caused by the HF diet when compared to the St diet (Fig. 13, Supplementary Fig. 5).

## Discussion

A number of studies have emphasized a link between obesity and central neuroinflammation leading to neurodegeneration in experimental rodent models and humans (reviewed in [2]). In this study, an age-dependent effect of HF diet-induced obesity on potential brain neuroinflammation in THY-Tau22 mice, a model of tau pathology, was studied separately in males and females.

Open field data in this study suggested that just the impact of the higher age of mice was the strongest factor for lower mobility and higher anxiety, which was deepened in females by THY-Tau22 genotype and the HF diet. Regarding memory, THY-Tau22 males showed impaired long-term spatial memory in the Morris water maze at 9 months [9] and from 9 to 10 months of age [7]. The Y-maze test detected impaired short-term spatial memory in 12- to 13-month-old THY-Tau22 males compared to wt controls [8]. The current study showed significantly impaired short-term spatial memory in the Y-maze test in 7- and 11-month-old THY-Tau22 males but not females compared to the respective wt controls. The decrease in short-term memory was in line with the increase in neuroinflammation, as discussed below, affecting more males than females and pointed to neuroinflammation as a possible cause of the memory deficit.

The body weight of THY-Tau22 mice fed the St diet was significantly lower than that of wt mice on St diet as expected [16]. Nevertheless, our other results showed the same trend for HF diet-fed males, but not females, where the body weight of THY-Tau22 females increased more than wt females on the HF diet. However, HF diet-induced obesity increased the weight of SCAT and IPAT and plasma cholesterol in 7- and 11-month-old mice and liver weight and plasma FFA in 11-month-old mice, and all these data pointed to the development of IR. In humans, the development of IR in muscle and the liver was linked to FFA originating mainly from excessive subcutaneous fat [30]. HOMA-IR and QUICKI indexes confirmed significant peripheral IR in all mice with HF diet-induced obesity in our study. Similarly, in our previous studies in C57BL/6 mice strain, the background for THY-Tau22 mice, after feeding the HF diet for several months we repeatedly registered not only very significant obesity but also glucose intolerance and an enhanced HOMA-IR index, which was more distinct in males [18, 23, 31]. However, our current study did not show different tolerances to glucose (tested by OGTT) among the 7- or 11-month-old groups. Moreover, in this study, only males but not females 11-month-old developed massive liver steatosis in the fatty liver as another result of IR. However, a previous study by others [16] reported through an insulin tolerance test that 20 weeks of HF-diet feeding did not attenuate peripheral sensitivity to insulin in THY-Tau22 males at 7 months of age. Furthermore, the HF diet-induced obesity led to the higher plasma inflammation demonstrated by an increased level of CRP. Several studies proved the direct link between the amount of body adipocytes which produce proinflammatory cytokines and increased body inflammation in rodents. Such peripheral inflammation could be associated with the central inflammation (reviewed in [2]). However, the present study showed that C57Bl/6 J mouse controls fed 5 or 9 months of HF diet did not show the direct link between peripheral and central inflammation, neither in males nor in females. A similar situation was observed in THY-Tau22 females, but not males. These results could suggest that in these two particular mouse models, C57Bl/6 J and THY-Tau22, there is no direct link between peripheral and central inflammation, or it could occur only in THY-Tau22 males from the age of 7 months.

Regarding central insulin sensitivity, surprisingly, an enhanced anorexigenic response to centrally administered insulin was reported in THY-Tau22 males 8 to 10 months old with obvious tau pathology and memory deficits [32]. In addition, hippocampal insulin signaling was reported to be increased by the HF diet in 7-month-old THY-Tau22 males [16]. In accordance, 11-month-old THY-Tau22 males fed a St diet showed increased activation of kinases implicated in the insulin signaling cascade as exhibited by increased phosphorylation of PDK-1 at residue S241, Akt at both epitopes T308 and S473, and increased inhibition of GSK-3β kinase activity toward tau protein exhibited by increased phosphorylation at S9 residue in hippocampi compared to wt males of the same age. However, in our study, 11-month-old THY-Tau22 males that were 9 months on the HF diet displayed decreased phosphorylation of pPDK-1 and pAkt at T308, which is directly phosphorylated by pPDK-1. On the other hand, decreased phosphorylation was not observed on Akt at S473; nevertheless, this epitope can be phosphorylated by different kinases such as the mTORC2 complex (mammalian target of rapamycin complex 2) [33]. The impact of the HF diet on the phosphorylation of GSK-3β at S9 residue of 11-month-old THY-Tau22 mice of both sexes was not observed.

However, the activity of another tau kinase, ERK1/2, was significantly increased in 11-month-old THY-Tau22 females fed the HF diet compared to St diet-fed females. The HF diet significantly decreased the inactivated form of PP2A, the main tau phosphatase, pPP2A C (Y307), in THY-Tau22 males. No further impact of the HF diet on PP2A activity was observed.

Overall, the peripheral IR could be defined by increased HOMA and decreased QUICKI indexes. To define the central IR could be more complex and it requires careful interpretation. In this study, we pointed to the differences and changes in mouse hippocampi which could suggest some changes in brain insulin signaling.

In the first study on THY-Tau22 male mice, mild astrocytosis representing brain neuroinflammation was reported [7]. Another study demonstrated brain neuroinflammation in THY-Tau22 mice, shown in the mixture of both sexes, to be triggered by the microglia-originated chemokine CCL3 and to progress with the age of animals [10]. In our study, the progression of microgliosis demonstrated through Iba1 immunohistochemistry was quite different in males and females. In THY-Tau22 males but not females, microgliosis progressed with the age of the animals and was enormously excited by the HF diet. The HF diet increased the amount of clusters of microglia (marked with Iba1) in the hippocampus, cortex, and amygdala of 11-month-old THY-Tau22 males massively and wt moderately. Previously, HF-diet feeding for 3 months was found to cause an increased incidence of hippocampal Iba1-positive (Iba1+) microglia in 5-month-old males of the C57Bl/6 J strain, which is the basis of THY-Tau22 [34]. A larger effect of 3 months of HF-diet feeding on hippocampal Iba1+ was reported in males but not females in 5-month-old C57Bl/6 J mice [35]. The WB of hippocampal anti-CD11b antibody showed only one significant increase in 7-month-old transgenic females on the HF diet when compared to wt females on the same diet. This finding could be caused by the fact that the CD11b antigen is expressed on leukocytes in general more than on microglia specifically. Analogously, with an increase in Iba1+ microglia in this study, an enhanced incidence of GFAP-positive (GFAP+) reactive astrocytes was observed in the cortex of 11-month-old transgenic males but not females fed the HF diet. This outcome corresponded with the fact that an increase in Iba1+ microglia was observed only in the amygdala of HF diet-fed THY-Tau22 females aged 11 months. This result suggests that neuroinflammation in older THY-Tau22 differed between the sexes and that HF diet-induced obesity led to worsened neuroinflammation in males.

Previous studies showed a higher incidence of activated microglia and reactive astrocytes, both markers of developing neuroinflammation, in close vicinity to the neurofibrillary tangles of hyper and abnormally phosphorylated tau protein (reviewed in [36]). Indeed, our results showed that a high number of reactive astrocytes colocalized with tau protein phosphorylated at the T231 residue in the hippocampus and amygdala in THY-Tau22 males and females on both the St and HF diets. A lower abundance of colocalized astrocytes and pTau (T231) was also observed in the cortex. The pTau T231 residue was found to engage selectively in a salt bridge with neighboring R230 and to compete for the formation of intermolecular salt bridges to tubulin [37]. Similar results were observed previously in 12-month-old THY-Tau22 mice of both sexes, where a higher incidence of CD11b-positive (CD11b+) and GFAP+ glial cells was reported in the CA1 region of the hippocampus, colocalized with tau phosphorylated at residue S422 [10]. The pTau S422 residue was also suggested to play a role in tau protein aggregation and filament formation [38, 39]. Leboucher et al. showed increased hippocampal tau phosphorylation (S214, S404, and S422), which was due to 20 weeks of HF-diet feeding in 7-month-old THY-Tau22 males [16]. Even though our study showed increased pTau (T231) colocalized with activated astrocytes by IHC in THY-Tau22 animals when compared to wt animals, the current WB data did not indicate a significant increase in hippocampal tau phosphorylation (at T212, S214, T231, S396, S404, and S422) in THY-Tau22 animals because of HF-diet feeding even at 11 months of age, when the tau pathology was described to be fully developed [7]. However, the increase in pTau colocalized with astrocytes determined by IHC more clearly confirms its pathological effect arising from specific local changes than pTau determined by the WB in a lysate of the whole hippocampus.

In this study, a significant decrease in synaptic and postsynaptic plasticity, expressed by a lower incidence of both synaptophysin and PSD95, was observed only in 11-month-old THY-Tau22 females but not males on both diets compared to wt controls. This result pointed to sex differences in synaptic plasticity of THY-Tau22 mice and suggested that loss of synaptic plasticity could occur independently of neuroinflammation in THY-Tau22.

The THY-Tau22 mouse model is an artificial model of tau pathology with inserted mutated human tau protein. THY-Tau22 did not evince Aβ pathology and therefore did not fully reflect AD pathology in humans. Therefore, the results from this study should be carefully interpreted when human subjects or other animal model subjects are used in the study. However, a clear sex-dependent effect of HF diet-induced obesity on neuroinflammation in mice with mutated human tau was shown. Tau pathology is a main hallmark of human tauopathies, i.e., Pick’s disease, and one of the several of main hallmarks of human Alzheimer’s disease. Considering the fact that THY-Tau22 mouse model is an artificial model of tau pathology which could influence the overal disease expression, we suggest to consider the sex and overal fitness in age-related human studies related to brain neurodegeneration. Based on our results we propose that 7- to 11-month-old THY-Tau22 males fed both the St and HF diets represent the mouse model of tau pathology with accompanied neuroinflammation suitable for testing of possible therapeutic research. Furthermore, 11-month-old THY-Tau22 females fed both the St and HF diets represent the mouse model of tau pathology with accompanied worsened synapthogenesis suitable for testing of possible therapeutic research.

## Conclusions

To conclude, the presented results showed strong neuroinflammation represented by microgliosis (marked by Iba1) and astrocytosis (marked by GFAP) in 11-month-old THY-Tau22 males but not females. Such strong neuroinflammation was accompanied by massive tau protein phosphorylation at the T231 residue, which was mostly visible in the hippocampus and amygdala and moderately visible in the cortex. Furthermore, probably resulting from increased neuroinflammation in the brains of THY-Tau22 males, but not females, these males evinced impaired short-term spatial memory. Moreover, 11-month-old females, but not males, showed robustly worsened synaptic and postsynaptic plasticity even though they did not show worsened neuroinflammation caused by the tau transgene or HF diet. Such results indicated significant differences between the sexes of THY-Tau22 mice, and this finding should be kept in mind in future studies.

The HF diet caused a significant increase in body weight and related metabolic parameters, which led to peripheral IR in both transgenic and wt mice of both sexes. The present study linked the HF diet not only to peripheral IR but also to worsened neuroinflammation in the blood plasma of all HF-diet fed mice, and in the male THY-Tau22 brain.

## Supplementary Information

**Additional file 1: Fig. S1**. Behavioral test. Open field (n = 7-14). Data are presented as mean ± SEM and were statistically analyzed by Mann-Whitney t-test (*) within each age and sex group (*p < 0.05; **p < 0.01). The age comparison was performed by mixed-effects analysis and Bonferroni’s post hoc test (^#^). A value significance between 11 and 3, and 11 and 7 months in particular group at wall distance was ^#^p < 0.001 in all cases (not shown for clarity). **Fig. S2** Western blots of hippocampal microgliosis marker CD11b. (A) Western blots for specific proteins. (B) Quantification of (A) western blots. Data are presented as mean ± SEM and were statistically analyzed by Mann-Whitney t-test within each age and sex group (*p < 0.05; n = 6). Mouse wt group on St diet was set as 100 %. The intensity of all proteins was related to particular β-actin intensity. CD11b: cluster of differentiation molecule 11b known as integrin alpha M; Iba1: ionized calcium-binding adaptor molecule 1. **Fig. S3** Western blots of hippocampal insulin signaling pathway markers of 11-month-old THY-Tau22 and wt mice. (A) Western blots for specific proteins. (B) Quantification of (A) western blots. Data are presented as mean ± SEM and were statistically analyzed by Mann-Whitney t-test within each age and sex group (*p < 0.05; **p < 0.01; n = 6). Mouse wt group on St diet was set as 100 %. The intensity of all proteins was related to particular β-actin intensity. Akt: protein kinase B; GSK-3β: glycogen synthase kinase 3 beta; PDK-1: phosphoinositide-dependent kinase-1. **Fig. S4** Western blots of hippocampal ERK1/2 of 11-month-old THY-Tau22 and wt mice. (A) Western blots for specific proteins. (B) Quantification of (A) western blots. Data are presented as mean ± SEM and were statistically analyzed by Mann-Whitney t-test within each age and sex group (**p < 0.01; n = 6). Mouse THY-Tau22 group on St diet was set as 100 %. The intensity of all proteins was related to particular β-actin intensity. ERK1/2: extracellular signal regulated kinase. **Fig. S5** Western blots of hippocampal Tau5 of 11-month-old THY-Tau22 mice. (A) Western blots for specific proteins. (B) Quantification of (A) western blots. Data are presented as mean ± SEM and were statistically analyzed using Mann-Whitney t-test. No statistically significant difference was observed. (p ≥ 0.05; n = 6). Mouse THY-Tau22 group on St diet was set as 100 %. The intensity of Tau5 was related to particular β-actin intensity.

## Data Availability

The datasets generated during and/or analyzed during the current study are available from the corresponding author on reasonable request. The supplementary file including figures supporting this study is included in this published article.

## Abbreviations

Aβ: Beta-amyloid
AD: Alzheimer’s disease
Akt: Protein kinase B
pAkt: Akt phosphorylated at T308 and/or S473 residues
ANOVA: Analysis of variance
AUC_ΔGlucose_: Area under the curve calculated from OGTT
BIOCEV: Biotechnology and Biomedicine Centre of the Academy of Sciences and Charles University
BSA: Bovine serum albumin
CD11b: Cluster of differentiation molecule 11b known as integrin alpha M
CRP: C-reactive protein
DAB: 3,3′-diaminobenzidine
DAPI: 4′,6′-diamidin-2-phenylindole
ERK1/2: Extracellular signal-regulated kinase 1/2 known as mitogen-activated protein kinase, MAPK
pERK1/2 (T202/Y204): ERK1/2 phosphorylated at T202/Y204 residues
FFA: Free fatty acids
GFAP: Glial fibrillary acidic protein
GFAP+: GFAP positive
GSK-3β: Glycogen synthase kinase 3 beta
pGSK-3β (S9): GSK-3β phosphorylated at S9 residue
HF: High-fat diet
HOMA-IR: Homeostatic model assessment of insulin resistance
Iba1: Ionized calcium-binding adaptor molecule 1
Iba1+: Iba1 positive
IPAT: Intraperitoneal adipose tissue
IR: Insulin resistance
mAb: Monoclonal antibody
NZ: New zone in Y-maze test
NGS: Normal goat serum
OGTT: Oral glucose tolerance test
pAb: Polyclonal antibody
PDK-1: Phosphoinositide-dependent kinase-1
pPDK-1 (S241): PDK-1 phosphorylated at S241 residue
PI3Kp85: p85 regulatory subunit of phosphoinositide 3-kinase
PP2A: Protein phosphatase 2A
met-PP2A C (L309): PP2A C subunit methylated at L309 residue
pPP2A C (Y307): Phosphorylated PP2A C subunit at Y307 residue
QUICKI: Quantitative insulin sensitivity check index
PSD95: Postsynaptic density protein 95
SCAT: Subcutaneous adipose tissue
SEM: Standard error of mean
St: Standard diet
pTau: Phosphorylated tau protein
Tau22 and THY-Tau22: THY-Tau22 genotype
TG: Triglyceride
W: Week
WB: Western blots
wt: Wild type
