# Supplementary material for: Aging and high-fat diet feeding lead to peripheral insulin resistance and sex-dependent changes in brain of mouse model of tau pathology THY-Tau22
Source: J Neuroinflammation. 2021 Jun 22;18:141. doi: 10.1186/s12974-021-02190-3 (PMC8218481; doi:10.1186/s12974-021-02190-3)
Supplement: Supplementary file 1 — Additional file 1: Fig. S1. Behavioral test. Open field (n = 7-14). Data are presented as mean ± SEM and were statistically analyzed by Mann-Whitney t-test (*) within each age and sex group (*p < 0.05; **p < 0.01). The age comparison was performed by mixed-effects analysis and Bonferroni’s post hoc test (#). A value significance between 11 and 3, and 11 and 7 months in particular group at wall distance was #p < 0.001 in all cases (not shown for clarity). Fig. S2 Western blots of hippocampal microgliosis marker CD11b. (A) Western blots for specific proteins. (B) Quantification of (A) western blots. Data are presented as mean ± SEM and were statistically analyzed by Mann-Whitney t-test within each age and sex group (*p < 0.05; n = 6). Mouse wt group on St diet was set as 100 %. The intensity of all proteins was related to particular β-actin intensity. CD11b: cluster of differentiation molecule 11b known as integrin alpha M; Iba1: ionized calcium-binding adaptor molecule 1. Fig. S3 Western blots of hippocampal insulin signaling pathway markers of 11-month-old THY-Tau22 and wt mice. (A) Western blots for specific proteins. (B) Quantification of (A) western blots. Data are presented as mean ± SEM and were statistically analyzed by Mann-Whitney t-test within each age and sex group (*p < 0.05; **p < 0.01; n = 6). Mouse wt group on St diet was set as 100 %. The intensity of all proteins was related to particular β-actin intensity. Akt: protein kinase B; GSK-3β: glycogen synthase kinase 3 beta; PDK-1: phosphoinositide-dependent kinase-1. Fig. S4 Western blots of hippocampal ERK1/2 of 11-month-old THY-Tau22 and wt mice. (A) Western blots for specific proteins. (B) Quantification of (A) western blots. Data are presented as mean ± SEM and were statistically analyzed by Mann-Whitney t-test within each age and sex group (**p < 0.01; n = 6). Mouse THY-Tau22 group on St diet was set as 100 %. The intensity of all proteins was related to particular β-actin intensity. ERK1/2: extrace [file 12974_2021_2190_MOESM1_ESM.docx]

**Supplementary figures**


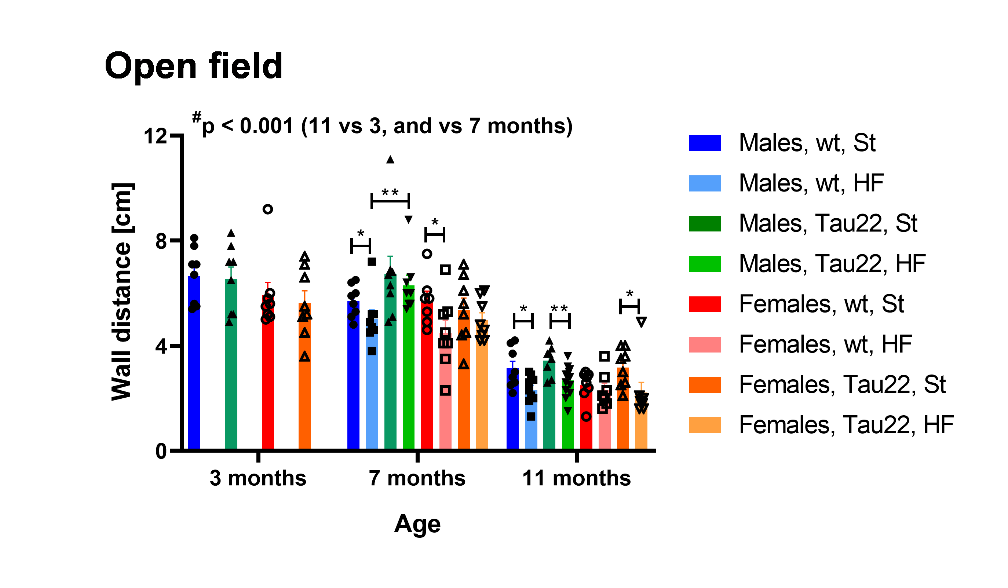


Supplementary Fig. 1 Behavioral test. Open field (n = 7-14). Data are presented as mean ± SEM and were statistically analyzed by Mann-Whitney t-test (*) within each age and sex group (*p < 0.05; **p < 0.01). The age comparison was performed by mixed-effects analysis and Bonferroni’s post hoc test (^#^). A value significance between 11 and 3, and 11 and 7 months in particular group at wall distance was ^#^p < 0.001 in all cases (not shown for clarity).


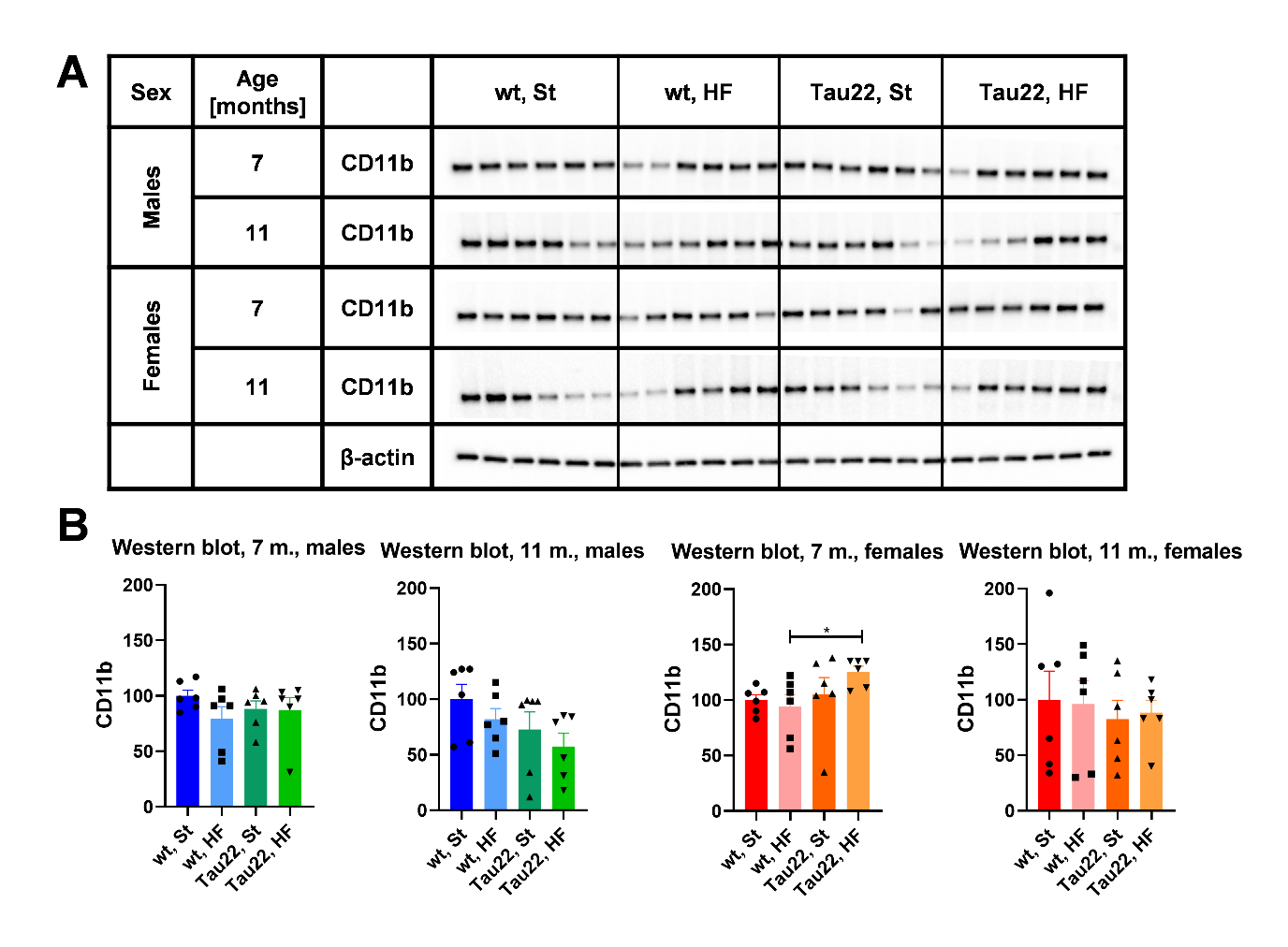


Supplementary Fig. 2 Western blots of hippocampal microgliosis marker CD11b. (A) Western blots for specific proteins. (B) Quantification of (A) western blots. Data are presented as mean ± SEM and were statistically analyzed by Mann-Whitney t-test within each age and sex group (*p < 0.05; n = 6). Mouse wt group on St diet was set as 100 %. The intensity of all proteins was related to particular β-actin intensity. CD11b: cluster of differentiation molecule 11b known as integrin alpha M; Iba1: ionized calcium-binding adaptor molecule 1.


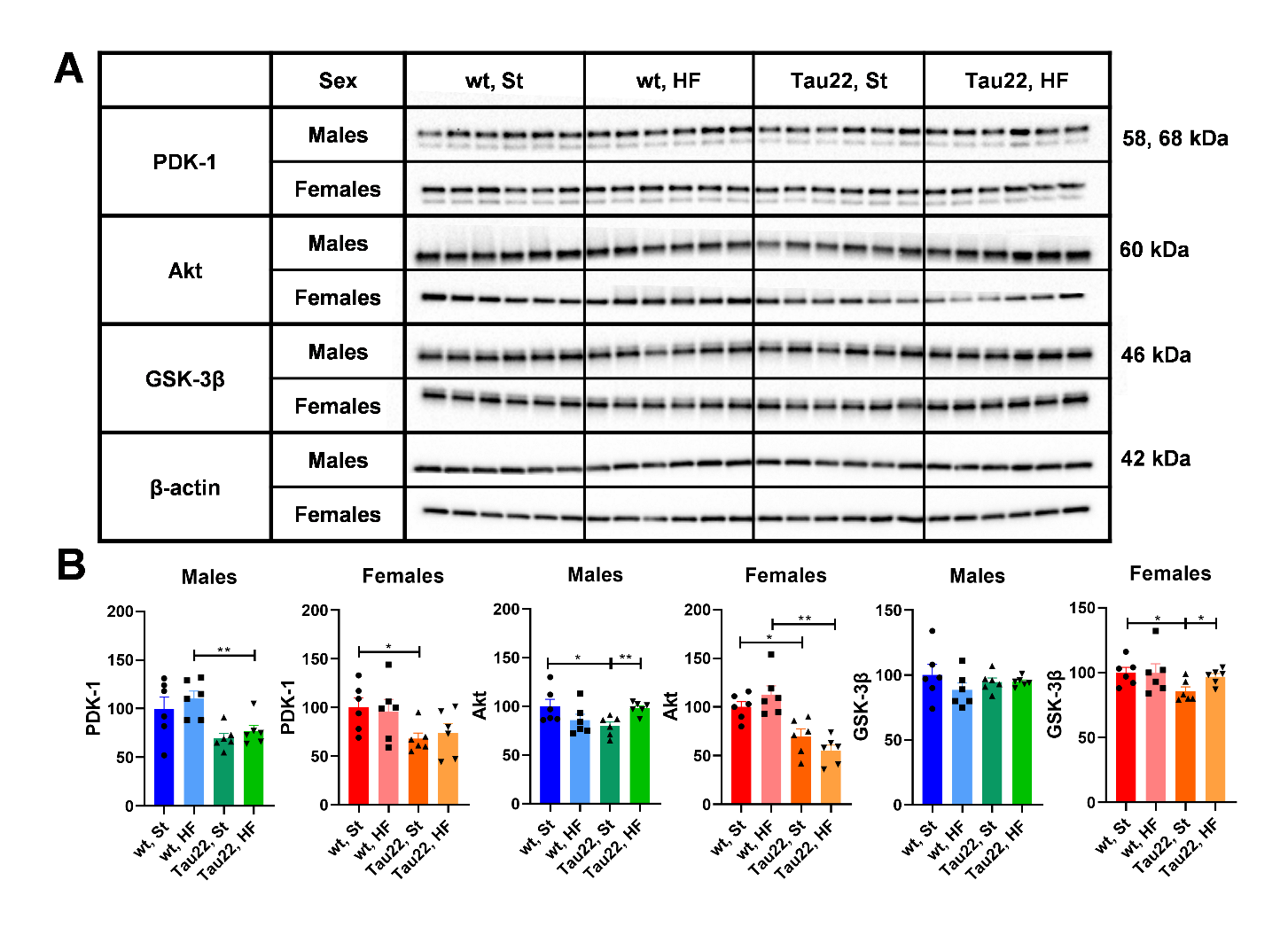


Supplementary Fig. 3 Western blots of hippocampal insulin signaling pathway markers of 11-month-old THY-Tau22 and wt mice. (A) Western blots for specific proteins. (B) Quantification of (A) western blots. Data are presented as mean ± SEM and were statistically analyzed by Mann-Whitney t-test within each age and sex group (*p < 0.05; **p < 0.01; n = 6). Mouse wt group on St diet was set as 100 %. The intensity of all proteins was related to particular β-actin intensity. Akt: protein kinase B; GSK-3β: glycogen synthase kinase 3 beta; PDK-1: phosphoinositide-dependent kinase-1.


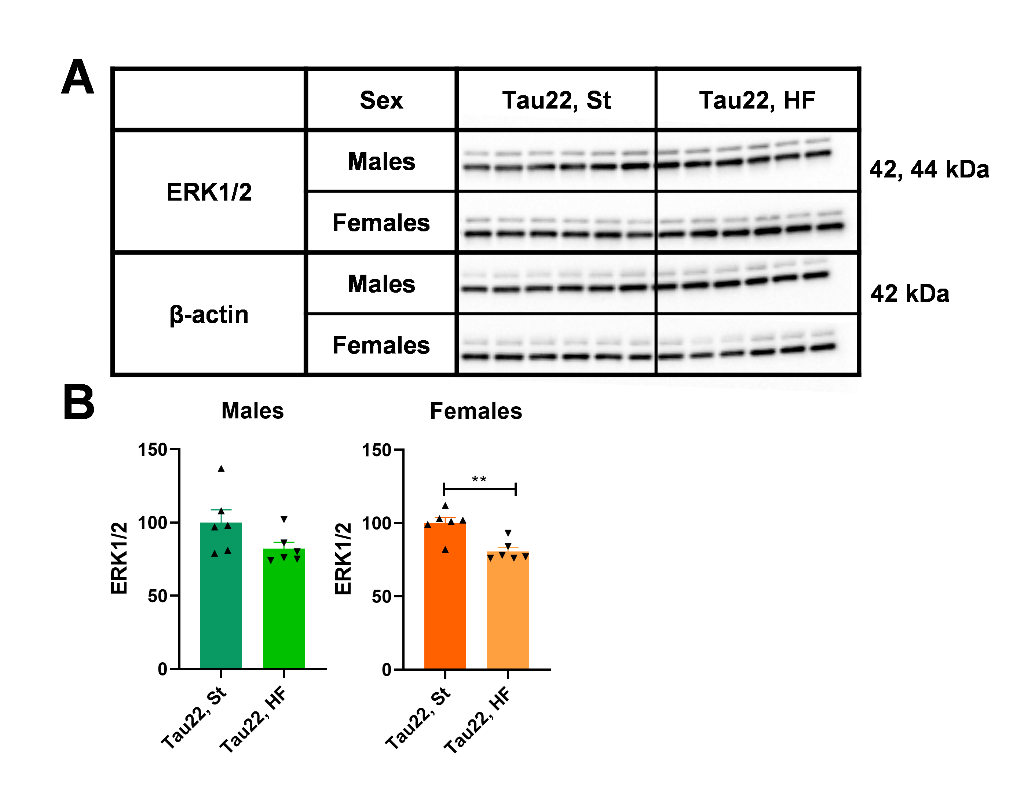


Supplementary Fig. 4 Western blots of hippocampal ERK1/2 of 11-month-old THY-Tau22 and wt mice. (A) Western blots for specific proteins. (B) Quantification of (A) western blots. Data are presented as mean ± SEM and were statistically analyzed by Mann-Whitney t-test within each age and sex group (**p < 0.01; n = 6). Mouse THY-Tau22 group on St diet was set as 100 %. The intensity of all proteins was related to particular β-actin intensity. ERK1/2: extracellular signal regulated kinase.


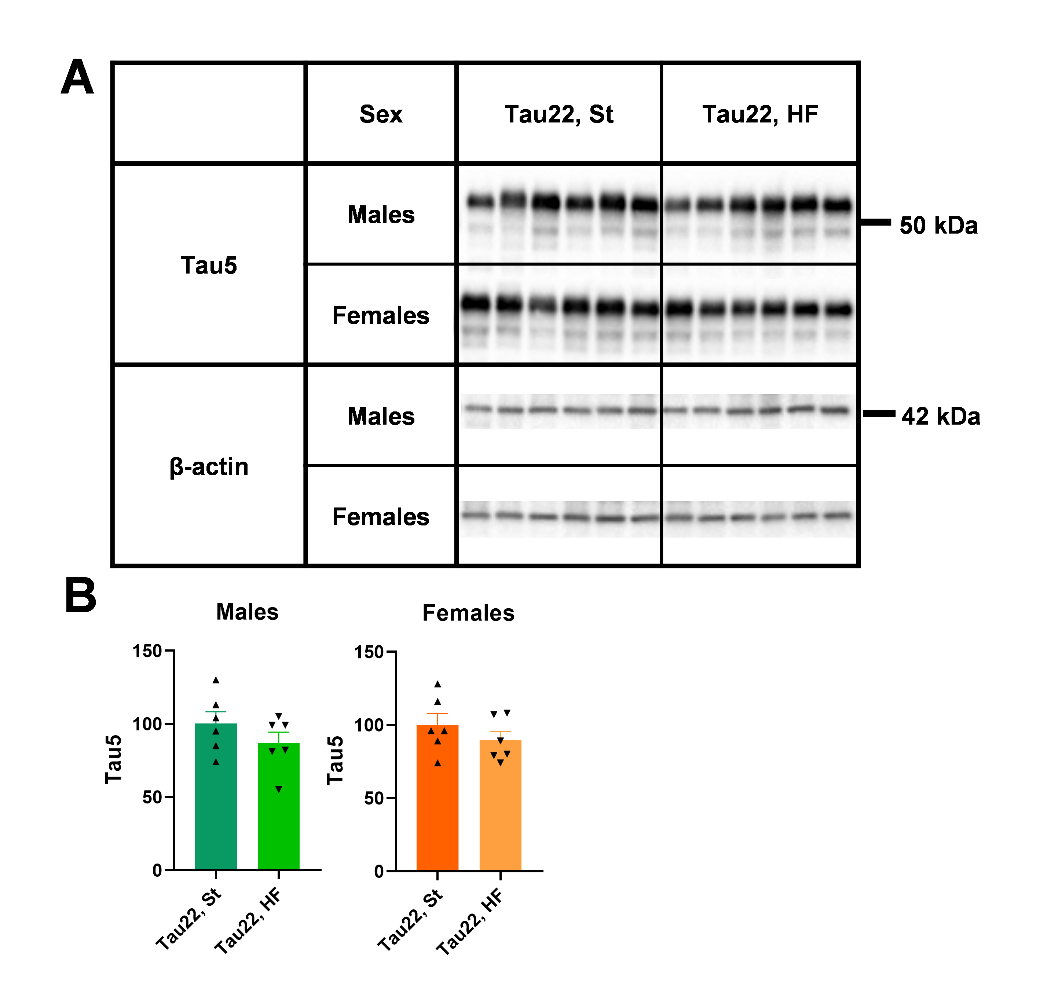


Supplementary Fig. 5 Western blots of hippocampal Tau5 of 11-month-old THY-Tau22 mice. (A) Western blots for specific proteins. (B) Quantification of (A) western blots. Data are presented as mean ± SEM and were statistically analyzed using Mann-Whitney t-test. No statistically significant difference was observed. (p ≥ 0.05; n = 6). Mouse THY-Tau22 group on St diet was set as 100 %. The intensity of Tau5 was related to particular β-actin intensity.
